# Hic-5 regulates epithelial to mesenchymal transition in ovarian cancer cells in a TGFβ1-independent manner

**DOI:** 10.18632/oncotarget.19714

**Published:** 2017-07-31

**Authors:** Razan Sheta, Zhi-Qiang Wang, Magdalena Bachvarova, Marie Plante, Jean Gregoire, Marie-Claude Renaud, Alexandra Sebastianelli, Stephane Gobeil, Chantale Morin, Elizabeth Macdonald, Barbara Vanderhyden, Dimcho Bachvarov

**Affiliations:** ^1^ Department of Molecular Medicine, Université Laval, Québec, Québec, Canada; ^2^ Centre de recherche du CHU de Québec, L’Hôtel-Dieu de Québec, Québec, Québec, Canada; ^3^ Department of Obstetrics and Gynecology, Université Laval, Québec, Québec, Canada; ^4^ Centre de recherche du CHU de Québec, CHUL, Québec, Québec, Canada; ^5^ Department of Cellular and Molecular Medicine, University of Ottawa, Ottawa, Ontario, Canada

**Keywords:** Hic-5, TGFB1I1, epithelial ovarian cancer, epithelial-to-mesenchymal transition, RhoA/ROCK

## Abstract

The molecular basis of epithelial ovarian cancer (EOC) dissemination is still poorly understood. We have previously identified the hydrogen peroxide-inducible clone-5 (Hic-5) gene as hypomethylated in high-grade (HG) serous EOC tumors, compared to normal ovarian tissues. Hic-5 is a focal adhesion scaffold protein and has been primarily studied for its role as a key mediator of TGF-β–induced epithelial-to-mesenchymal transition (EMT) in epithelial cells of both normal and malignant origin; however, its role in EOC has been never investigated.

Here we demonstrate that Hic-5 is overexpressed in advanced EOC, and that Hic-5 is upregulated upon TGFβ1 treatment in the EOC cell line with epithelial morphology (A2780s), associated with EMT induction. However, ectopic expression of Hic-5 in A2780s cells induces EMT independently of TGFβ1, accompanied with enhancement of cellular proliferation rate and migratory/invasive capacity and increased resistance to chemotherapeutic drugs. Moreover, Hic-5 knockdown in the EOC cells with mesenchymal morphology (SKOV3) was accompanied by induction of mesenchymal-to-epithelial transition (MET), followed by a reduction of their proliferative, migratory/invasive capacity, and increased drugs sensitivity *in vitro*, as well as enhanced tumor cell colonization and metastatic growth *in vivo*. The modulation of Hic-5 expression in EOC cells resulted in altered regulation of numerous EMT-related canonical pathways and was indicative for a possible role of Hic-5 in controlling EMT through a RhoA/ROCK mediated mechanism.

To our knowledge, this is the first report examining the role of Hic-5 in EOC, and its role in maintaining the mesenchymal phenotype of EOC cells independently of exogenous TGFβ1 treatment.

## INTRODUCTION

Epithelial ovarian cancer (EOC) accounts for 4% of all cancers in women and is the leading cause of death from gynecologic malignancies [1]. Despite treatment improvements, long-term survival rates for patients with advanced disease remain disappointing [2]. The molecular basis of EOC initiation and progression is still poorly understood. To establish novel therapeutic and diagnostic strategies against this deadly disease, it is essential to understand its molecular pathology.

Similar to other malignancies, aberrant DNA methylation, including global hypomethylation of heterochromatin and local CpG island methylation, occurs in EOC and contributes to ovarian tumorigenesis and mechanisms of chemoresistance [3]. Applying a more global array-based technology, several studies have demonstrated that DNA methylation changes in EOC are cumulative with disease progression and chemotherapy (CT) resistance [4–6]. Using a similar approach (methylated DNA immunoprecipitation coupled to CpG island tiling arrays) we have recently shown that DNA hypermethylation occurs in less invasive/early stages of ovarian tumorigenesis, while advanced disease was associated with DNA hypomethylation of a number of oncogenes, implicated in cancer progression, invasion/metastasis and probably chemoresistance [7]. The hydrogen peroxide-inducible clone-5 (Hic-5 gene, also known as TGFB1I1; ARA55; HIC5; and TSC-5) was among the genes identified to be notably hypomethylated in both low-malignant potential (LMP) and high grade (HG) serous EOC tumors [7]. The Hic-5 gene is a member of the paxillin superfamily of focal adhesion adaptor proteins [8]. The proteins of this family function as molecular scaffolds to regulate focal adhesion dynamics and actin cytoskeleton remodeling during cell migration [9]. Focal adhesion complexes work to coordinate the maintenance of the mesenchymal phenotype characterized by elongated fibroblast like morphology [7, 10, 11]. Interestingly, the Hic-5 protein has been found to be highly overexpressed in mesenchymal, fibroblastic and osteoblastic cell lines [12, 13], while displaying low expression in cell lines of epithelial origin [13–15]. The Hic-5 gene was originally identified upon screening for hydrogen peroxide, and TGFβ1- inducible genes in mouse osteoblast MC3T3-E1 cells [8]. Multiple studies have shown that TGFβ1 stimulation can actually induce Hic-5 expression in epithelial cells of different origins [15–18], but the exact relationship between Hic-5 function in cancer cells and its link to the TGFβ1- signal transduction pathway remains unclear. Nonetheless, several reports indicate that Hic-5 is upregulated not only in mesenchymal cells, but can actually get upregulated during the induction of the epithelial to mesenchymal transition (EMT) process, as this upregulation was shown to be essential in establishing the mesenchymal phenotype of certain epithelial cell lines [14, 19].

It is important to note that TGFβ1 stimulates not only Hic-5 expression, but also directly induces a number of other signaling cascades and kinases, including EMT [20, 21]. The Rho family of small GTPases, and the RhoA Kinase (ROCK) have been shown to have a direct impact on cytoskeletal remodeling pathways such as EMT [18, 22–26], and recent evidence points that Hic-5 expression can modulate the role of this kinase family during EMT [7, 12, 17].

EMT is also associated with the activation of different members of the Smad gene family [25, 27, 28], as Hic-5 was shown to interact with Smad proteins by shuttling into the nucleus and acting as a transcriptional coactivator of the androgen and glucocorticoid receptors [29]. Indeed, Hic-5 was previously reported to mediate certain TGFβ1-induced transcriptional mechanisms through a direct interaction with Smad3, Smad2 and Smad7 [30].

All these reports point towards a distinct role of the Hic-5 gene in modulating EMT, which in turn could directly impact the metastatic potential of cancer cells. Indeed, a number of studies have suggested for an oncogenic role of Hic-5 in different cancer types including prostate, breast and melanoma [31–33]. EMT has been also extensively studied for its role in EOC progression and metastasis [34–36], as EMT has been frequently associated with disease spreading and poor survival of EOC patients [37, 38]. The direct correlation between the process of EMT and EOC is also supported by studies examining the role of several EMT-associated genes (including the transcription factors Snail and Twist) and their metastatic potential in EOC tumors [39]. Despite these findings, the molecular mechanisms that actually direct EMT, and which specifically sustain the mesenchymal phenotype and thus the metastatic potential of EOC cells, have not been well investigated.

Since the role of Hic-5 in ovarian carcinogenesis has never been studied, we decided to examine whether Hic-5 is functionally implicated in EOC tumorigenesis including disease progression and response to treatment, and if it plays an integral role in EMT-associated events in EOC. Here we present experimental data, indicative for strong Hic-5 overexpression in HG serous EOC tumors, which probably correlates with its hypomethylated status. Moreover, modulation of Hic-5 expression in EOC cells with either epithelial or mesenchymal phenotype was strongly indicative for its direct (TGFβ1-independent) implication in EMT regulation, and more specifically, for its role in maintaining the mesenchymal phenotype of EOC cells. We also show that Hic-5 directs the EMT process through interacting with the RhoA/ROCK pathway. Our data also support our and others previous findings regarding the superior capacity of epithelial cancer cells in the metastatic colonization of distant sites, compared to cancer cells with mesenchymal-like morphology.

## RESULTS

### Analysis of Hic-5 expression in EOC tumors

We initially evaluated Hic-5 protein expression by immunohistochemistry (IHC) in numerous serous EOC tumors and ovarian normal tissue samples, using tissue microarrays (TMAs). Our TMAs contained triplicate cores of 130 tissue samples, comprising 13 LMP serous tumors, 104 HG serous ovarian tumors and 13 normal ovarian tissue samples as controls. Table 1 shows the major clinical characteristics of these patients for whom extensive follow-up clinical data (up to 5-years) were available. The age ranged from 41 to 83 years (median: 66 years). HG tumors were all grade 3 (100%) including stage III (69%) and stage IV (31%) tumors. The majority of patients (93%) received a combination treatment of platinum and paclitaxel. The median baseline CA125 was around 800. Forty percent of the patients had a progression or a recurrence within the first 6 months of follow-up; for 39% of the patients the progression-free survival (PFS) interval was in the range of 7 to 24 months, and 21% of the patients displayed PFS values higher than 25 months (Table 1).

**Table 1:** Detailed patients’ clinicopathological characteristics

Variable	Range	n/total	%
Age (years)	≥65	64/130	49
	<65	66/130	51
Tissue/tumor type	Normal	13/130	10
	LMP	13/130	10
	High-grade	104/130	80
Grade	3	104/104	100
Stage	III	72/104	69
	IV	32/104	31
Chemotherapy	platinum+taxol	97/104	93
	Other	7/104	7
CA125	≥800	47/104	45
	<800	57/104	55
PFS (months) *	0-6	41/103	40
	7-24	40/103	39
	> 25	22/103	21

*Extended follow-up, including PFS values, were available for 103 patients.

**Figure 1:**
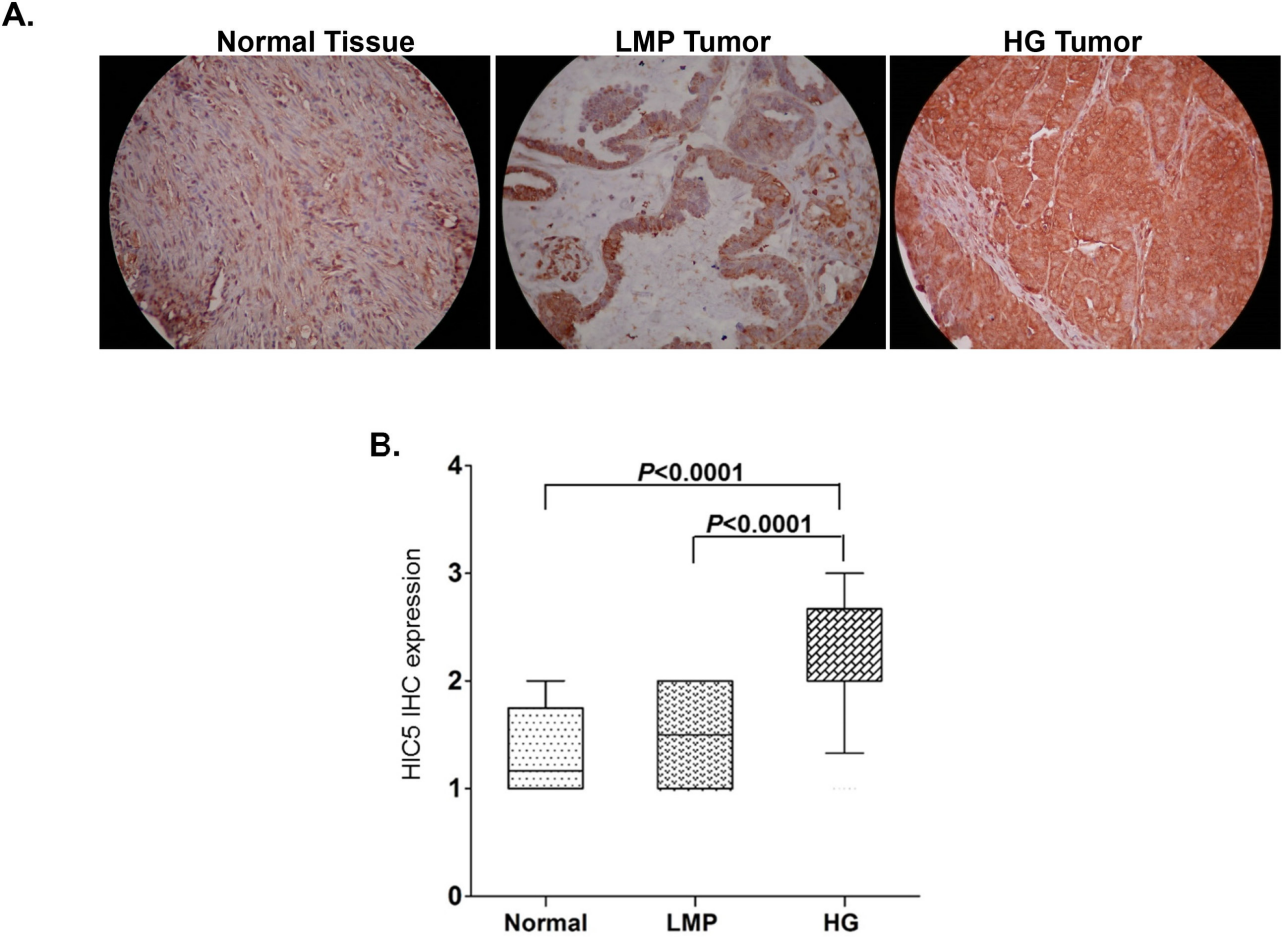
Analysis of Hic-5 expression in serous EOC tumors by IHC **(A)** Representative IHC images of Hic-5 protein expression in normal ovarian tissues, low-malignant potential (LMP) tumors and high-grade (HG) tumors. **(B)** Box-plot presentation of Hic-5 protein expression levels in normal ovarian tissues, low-malignant potential (LMP) tumors and high-grade (HG) tumors.

As seen from Figure 1A and 1B, Hic-5 displayed significantly higher expression in HG serous EOC tumors, when compared to normal tissues (*p* < 0.0001) and LMP tumors (*p* < 0.0001). This was also confirmed by analysing the Hic-5 protein expression levels in two human ovarian surface epithelial (HOSE) cell lines, which displayed very weak/lack of Hic- 5 expression, compared to the majority of the EOC cell lines analyzed (see Supplementary Figure 1A). We further constructed Kaplan–Meier survival curves based on the Hic-5 expression analyses in the cohort of 103 HG serous EOC patients. However, no significant relationship was found between higher Hic-5 expression and shorter PFS of serous EOC patients with advanced disease (*p* = 0.826; see Supplementary Figure 2A), which suggests that the staining intensity for Hic-5 in pre-treatment surgical EOC specimens is not predictive of PFS. Similarly, Hic-5 expression displayed no correlation with PFS and overall survival (OS) upon analyzing the TCGA, GEO and EGA datasets from 1287 EOC patients, accessible through the Kaplan Meier plotter Web portal (www.kmplot.com) [40] (see Supplementary Figure 2B and 2C).

### Hic-5 expression alterations in EOC cells point towards a direct (TGFβ1-independent) Hic-5 implication in establishing their mesenchymal phenotype

#### A. Hic-5 overexpression directs EMT in EOC cells with epithelial phenotype

**Figure 2:**
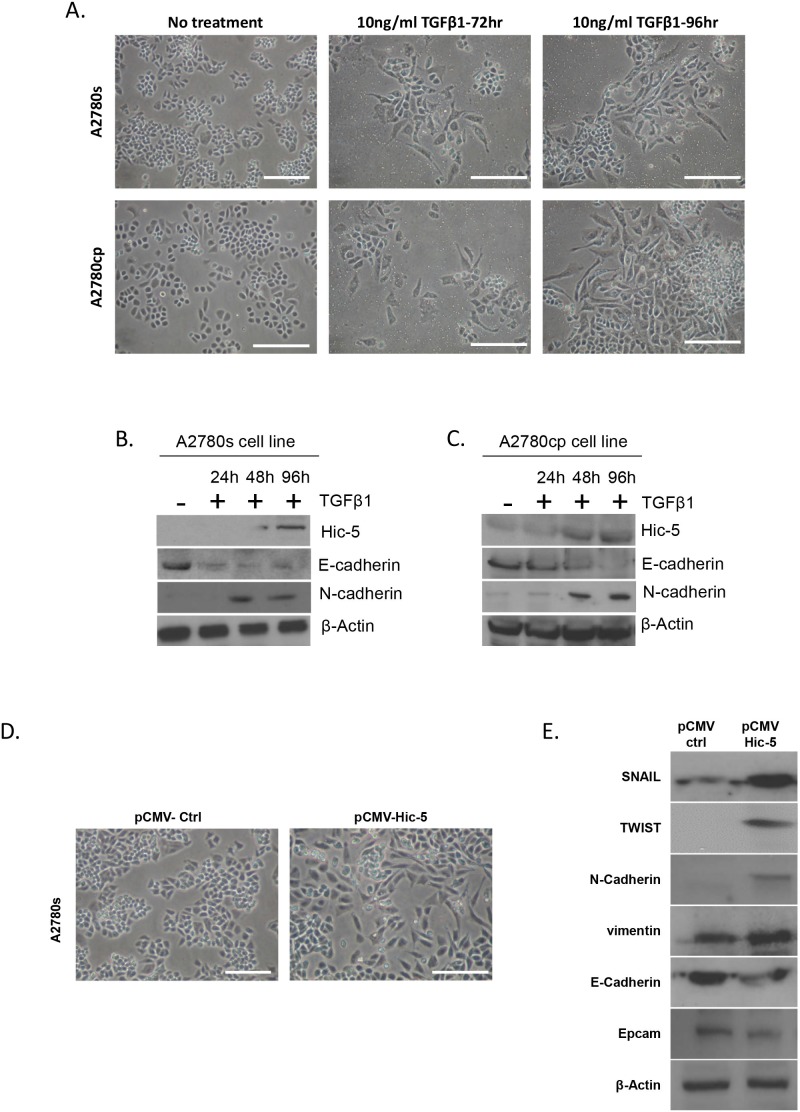
Effects of TGFβ1 treatment and Hic-5 ectopic expression on EMT modulation in EOC cells with epithelial phenotype **(A)** Representative phase-contrast images of A2780s and A2780cp cells before and after TGFβ1 treatment at 10 ng/ml after 72 and 96 hr. Scale Bar = 200 μm. **(B)** Western blot analysis of the expression of the Hic-5 gene in addition to the EMT markers in the cell line A2780s before and after treatment with 10 ng/ml of TGFβ1 at 24, 48, and 96 hr post-treatment. **(C)** Western blot analysis of the expression of the Hic-5 gene in addition to the EMT markers in the cell line A2780cp before and after treatment with 10 ng/ml of TGFβ1 at 24, 48, and 96 hr post-treatment. **(D)** Representative phase-contrast images of control clone (pCMV-Ctrl) and Hic-5 pCMV clone (pCMV-Hic-5). A2780s cells. Scale Bar = 200 μm. **(E)** Western blot analysis of the expression of different EMT markers in control clone (pCMV-Ctrl) and Hic-5 pCMV (pCMV-Hic-5) A2780s cells. β-actin was used as a loading control.

TGFβ1 is a well-known inducer of EMT, as evaluated in a variety of cell lines and various *in vitro* cell model systems [41]. Hic-5 was identified as a TGFβ1 inducible gene, which suggests a role for Hic-5 in the TGFβ1-mediated EMT regulation [8]. We tested several EOC cell lines for endogenous Hic-5 protein expression by Western blot analysis (see Supplementary Figure 1A). Among these, two EOC cell lines with epithelial phenotype (A2780s and A2780cp) displayed rather low endogenous Hic-5 protein expression. Prolonged TGFβ1 treatment of these cell lines resulted in the upregulation of the Hic-5 protein, which was mostly evident at day 4 compared to other shorter time points (Figure 2B and 2C). As previously shown [42], TGFβ1 treatment induced EMT in both A2780s and A2780cp cells, resulting in the acquisition of a mesenchymal (spindle-like) phenotype (Figure 2A), associated with the suppression of the epithelial marker E-cadherin, and strong expression of the mesenchymal marker N-cadherin (Figure 2B and 2C).

**Figure 3:**
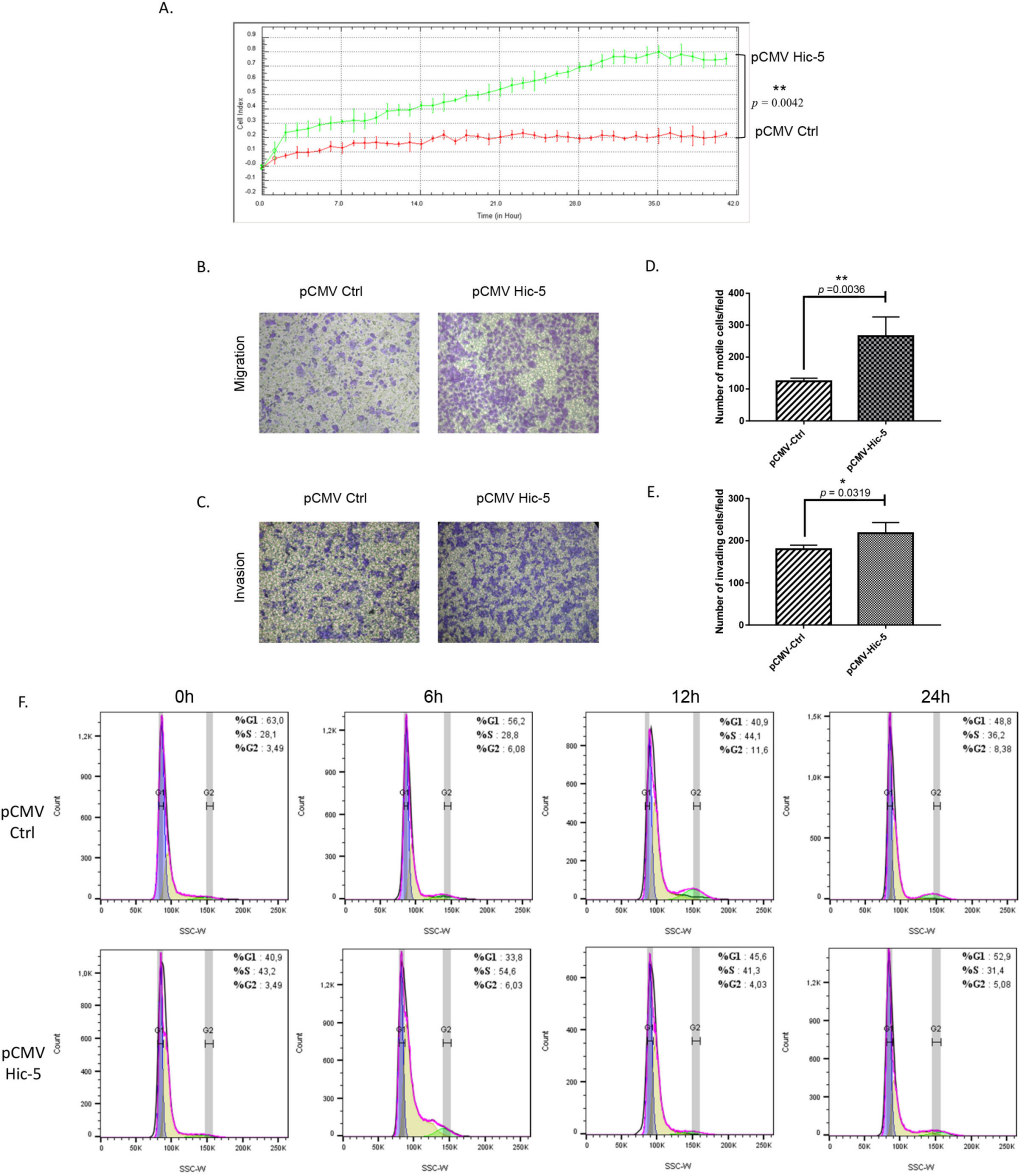
Effect of Hic-5 overexpression on A2780s cell proliferation migration, invasion and cell cycle **(A)** Cell proliferation of Hic-5 pCMV clone (pCMV-Hic-5) was compared to the control clone (pCMV-Ctrl). **(B)** Cell migration of Hic-5 pCMV clone (pCMV-Hic-5) was compared to the control clone (pCMV-Ctrl). Migration was assessed using Boyden-chamber assay. Cells from the Hic-5 pCMV clone (pCMV-Hic-5) and the control clone (pCMV-Ctrl) were seeded into the upper chambers in 0.1% FBS containing medium at a density of 1.5 × 10^4^ per well, and 600 μl of 1% FBS containing medium was placed in the lower chamber as a chemoattractant. After 24 hr incubation at 37°C in 5% CO_2_, the cells were fixed with cold methanol and stained with blue trypan solution. Migrated cells on the underside of the filter were photographed and counted by phase contrast microscopy. **(C)** Cell invasion was assayed in a similar way, as the upper chambers were coated with Matrigel. Here, NIH3T3 conditioned medium was added in the lower chamber as a chemoattractant (see Methods for details). All experiments were performed in triplicates. For each experiment, cell numbers were calculated as the total count from 10 random fields per filter (at magnification × 40). The bar graphs in panels **(D)** and **(E)** represent quantitative determinations of migration and invasion data obtained by selecting 10 random fields per filter under phase contrast microscopy and results are expressed as number of cell change (migration and invasion) of the Hic-5 pCMV (pCMV-Hic-5) clone compared to the control clone (pCMV-Ctrl). Differences between pCMV-Hic-5 and pCMV-Ctrl A2780s cells were determined by a Student's t-test; error bars denote mean ± SEM; (*p* < 0.05). **(F)** Cell-cycle profile was examined by flow cytometry and percentages of cells in G0/G1, S, and G2/M phase in the Hic-5 pCMV clone (pCMV-Hic-5) were compared to the control clone (pCMV Ctrl). Propidium iodide staining shows a decreased fraction of cells in the G1-phase and an increase accumulation of cells in the S-phase at 0 hr, and 6 hr post hydroxyurea removal in the pCMV-Hic-5 clone, when compared with the pCMV-Ctrl clone.

Next, we decided to verify if modulation of Hic-5 expression alone could exert any effect on EOC cellular phenotype and functional characteristics. Initially, we ectopically expressed the Hic-5 gene in A2780s cells, and the selection of one Hic-5 stably overexpressing clone (clone pCMV-Hic-5) was confirmed by Western blot analysis (Supplementary Figure 1B and 1C). The ectopic expression of Hic-5 in A2780s cells produced rather similar results to those observed after TGFβ1 treatment. Indeed, the Hic-5 overexpression induced EMT in A2780s cells (Figure 2D), associated with upregulation of the mesenchymal markers N-cadherin, TWIST, vimentin and SNAIL and reduction in the epithelial markers E-cadherin and EPCAM (Figure 2E). Moreover, the pCMV-Hic-5 A2780s cells showed significantly higher cell proliferation rates compared to control (Ctrl) cells (*p* = 0.0042) (Figure 3A), which was further supported by cell cycle analyses (Figure 3F). Indeed, the pCMV-Hic-5 A2780s cells exhibited a major increase in numbers at the S phase, with a decreased accumulation of cells in the G1 phase at 0 hr and 6 hr post-hydroxyurea removal (Figure 3F). Accordingly, Hic-5 overexpression in A2780s cells resulted in their significantly enhanced cell migration and invasion, when compared to Ctrl cells (Figures 3B-3E). Finally, Hic-5 overexpression also led to a major impact on A2780s cisplatin and paclitaxel sensitivity (drugs, conventionally used for first-line EOC CT), as pCMV-Hic-5 cells showed a significant decrease in sensitivity to the two drugs compared to the Ctrl (*p* = 0.0136, *p* = 0.0340) respectively (Supplementary Figure 1E).

#### B. Hic-5 suppression directs MET in EOC cells with mesenchymal phenotype

**Figure 4:**
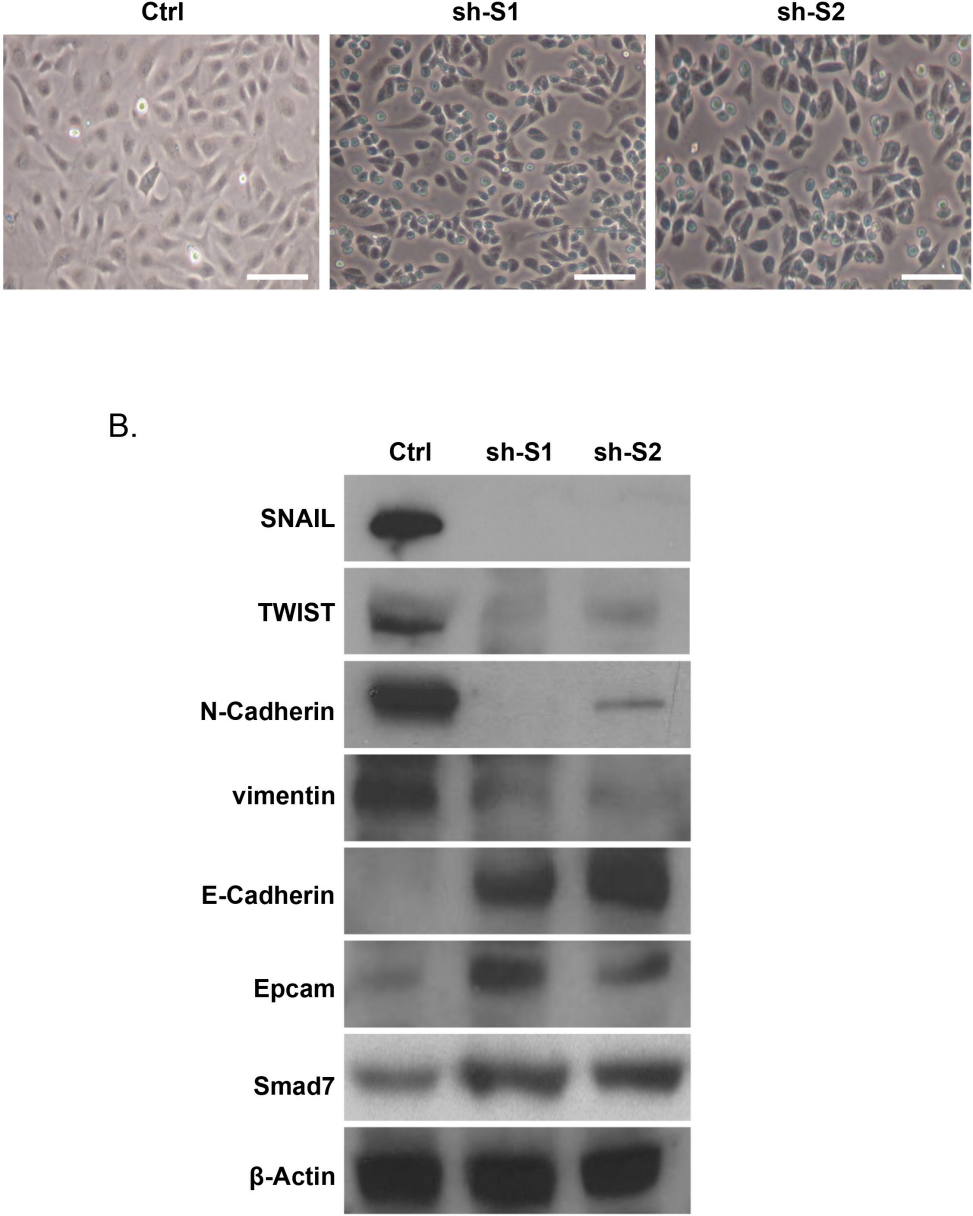
Hic-5 suppression directs MET in SKOV3 cells **(A)** Representative phase-contrast images of SKOV3 mock-transfected control (Ctrl) and shRNA-Hic-5 knockdown (KD) clones (sh-S1 and sh-S2). Scale Bar = 100 μm. **(B)** Western blot analysis of the expression of different EMT (epithelial and mesenchymal) markers, in addition to Smad7 in the control and the shRNA-Hic-5 knockdown (KD) SKOV3 clones. (sh-S1 and sh-S2). β-actin was used as a loading control.

**Figure 5:**
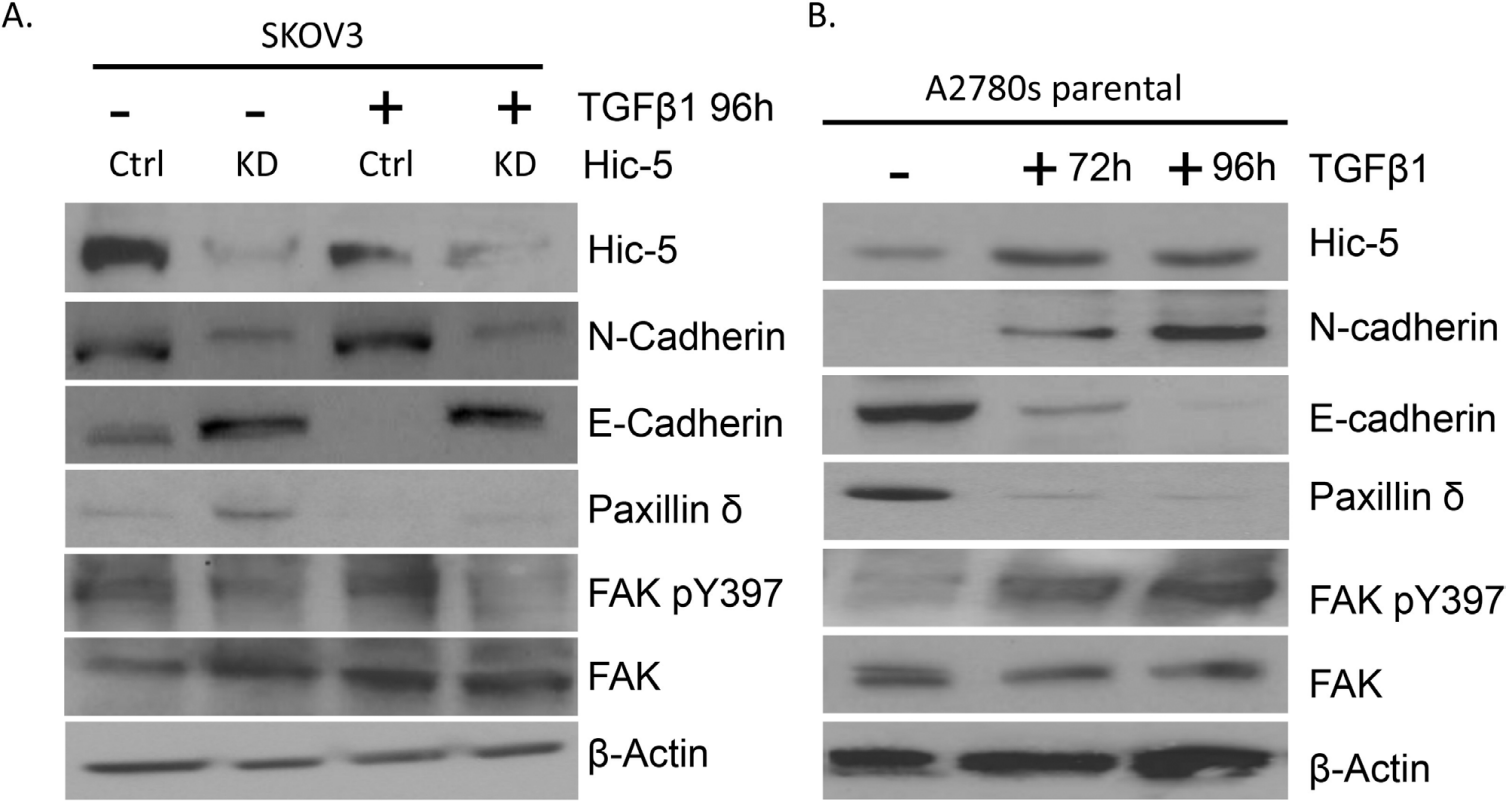
Effect of TGFβ1 treatment on Hic-5 knockdown and A2780s parental EOC cells **(A)** Western blot analysis of Hic-5 expression in addition to the EMT markers (N-cadherin and E-cadherin), Paxillin δ, FAK pY397, FAK in the SKOV3 mock-transfected control (Ctrl) and shRNA-Hic-5 knockdown clones (KD) treated (+) and untreated (-) with 5 ng/ml of TGFβ1 for 96 hr. **(B)** Western blot analysis of Hic-5 expression in addition to the EMT markers (N-cadherin and E-cadherin), Paxillin δ, FAK pY397, FAK in the A2780s parental cell line before and after treatment with 10 ng/ml of TGFβ1 at 72 hr and 96 hr. β-actin was used as a loading control.

The above data were suggestive for a possible direct role of Hic-5 in establishing the mesenchymal phenotype of EOC cells. This prompted us to verify if suppression of Hic-5 in mesenchymal type EOC cells could have an opposite (MET) effect. The mesenchymal-type EOC cell line SKOV3 displayed a strong endogenous Hic-5 expression (see Supplementary Figure 1A) and was initially used for shRNA-mediated suppression of Hic-5 expression. Stably transfected shRNA Hic-5 knockdown (KD) SKOV3 clones were consecutively selected by validation of Hic-5 mRNA/protein expression (Supplementary Figure 3A-3C). The Hic-5 suppression induced MET in the SKOV3 cell line, as both sh-S1 and sh-S2 SKOV3 clones exhibited a typical epithelial phenotype with cobblestone shaped cells, forming discrete clusters indicative for tight junctions (Figure 4A). This was further confirmed by analyzing the altered expression of specific EMT markers in both Ctrl and Hic-5 KD clones, as we observed a strong induction in the expression of the epithelial markers E-cadherin and EPCAM in the sh-S1 and sh-S2 SKOV3 clones compared to the Ctrl clone, while the expression levels of the mesenchymal markers N-cadherin, TWIST, SNAIL and vimentin were strongly down-regulated (Figure 4B). As expected, TGFβ1 treatment of Hic-5 KD SKOV3 clones did not result in Hic-5 induction due to the anti-Hic-5 shRNA expression; however and more importantly, TGFβ1 did not induce any phenotypic changes, as these cells retained their epithelial cobblestone morphology (data not shown) and maintained high E-cadherin and suppressed N-cadherin expression, similar to the non-treated Hic-5 KD SKOV3 clones (Figure 5A).

As also seen in Figure 4B, the Hic-5 KD in SKOV3 cells resulted in increased Smad7 expression. As previously reported, Smad7 can induce E-cadherin upregulation by an epigenetic mechanism, comprising DNA demethylation of the E-cadherin promoter region [43]. Indeed, methylation-specific PCR (MSP) analysis of the two DNA regions around E-cadherin ATG (start) codon (located at -292 nt to +2 nt and +61 nt to +262 nt) was indicative for DNA demethylation in these regions following Hic-5 suppression (Supplementary Figure 4), which confirms a possible Smad7-mediated epigenetic regulation of E-cadherin expression and its impact on MET, induced upon the downregulation of the Hic-5 gene.

Rather similar results were obtained when performing shRNA-mediated Hic-5 KD in the endometrioid EOC cell line TOV112, which also exhibits a mesenchymal-like phenotype and shows relatively high endogenous Hic-5 expression (Supplementary Figure 1A). We have generated two Hic-5 knockdown TOV112 clones sh-T1 and sh-T2 (Supplementary Figure 5A and 5B), which exhibited similar cellular morphology changes associated with the formation of typical cobblestone epithelial morphology (Supplementary Figure 5C), and accompanied with a strong overexpression of E-cadherin, EPCAM and the suppression of the mesenchymal markers N-cadherin, SNAIL, TWIST, and vimentin (Supplementary Figure 5D).

**Figure 6:**
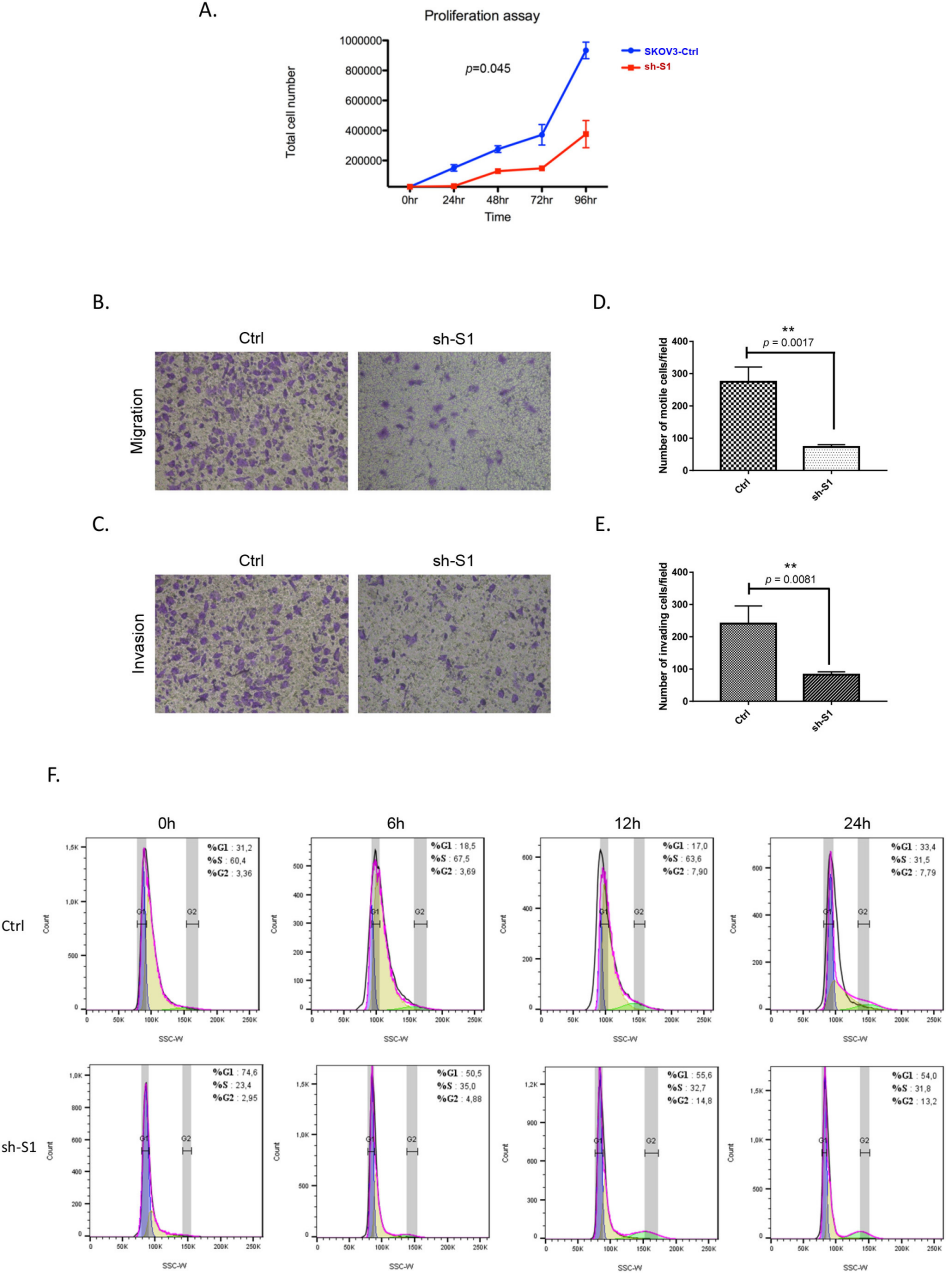
Effect of Hic-5 knockdown on SKOV3 cell proliferation migration, invasion and cell cycle **(A)** Cell proliferation of shRNA-Hic-5 knockdown (KD) clone (sh-S1) was compared to the mock-transfected control clone (Ctrl). **(B)** Cell migration of shRNA-Hic-5 knockdown (KD) clone (sh-S1) was compared to the control clone (Ctrl). Migration was assessed using Boyden-chamber assay (see legend of Figure 3 for details). **(C)** Cell invasion of shRNA-Hic-5 knockdown (KD) clone (sh-S1) was compared to the mock-transfected control clone (Ctrl); see legend of Figure 3 for details. The bar graphs in panels **(D)** and **(E)** represent quantitative determinations of migration and invasion data obtained by selecting 10 random fields per filter under phase contrast microscopy and results are expressed as number of cell change (migration and invasion) of the sh-S1 clone compared to the Ctrl clone. Differences between shRNA-Hic-5 knockdown clone (sh-S1) and mock-transfected SKOV3 control clone (Ctrl) were determined by a Student's t-test; error bars denote mean ± SEM; (*p* < 0.05). **(F)** Cell-cycle profile was examined by flow cytometry and percentages of cells in G0/G1, S, and G2/M phase in the shRNA-Hic-5 knockdown (KD) clone (sh-S1) were compared to the mock-transfected control clone (Ctrl). Propidium iodide staining shows an increased fraction of cells in the G1-phase and a decrease of cells in the S-phase at 0 hr, and especially at 6 hr and 12 hr post hydroxyurea removal in the (sh-S1) clone, when compared with Ctrl clone.

We subsequently investigated the impact of the Hic-5 gene suppression on SKOV3 cell proliferation, cell cycle control, migration, invasion, and sensitivity to cisplatin and paclitaxel. As seen from Figure 6A, the Hic-5 KD clone sh-S1 displayed a significantly lower proliferation rate compared to Ctrl cells (*p* = 0.045), which was further supported by colony formation assays, comparing the two clones sh-S1 and sh-S2 to Ctrl cells (*p* = 0.0023 and *p* = 0.0069 respectively; see Supplementary Figures 6A and 6B). The Hic-5 depletion also induced G1 cell cycle arrest (Figure 6F), which could explain the drastic reduction in the proliferation rates of these EOC cells observed earlier (Figure 6A). Additionally, Hic-5 KD SKOV3 cells exhibited significantly lower migration and invasion rates compared to Ctrl cells (*p* = 0.0017 and *p* = 0.0081 respectively; see Figures 6B-6E), which was also confirmed by a wound-healing assay (see Supplementary Figure 6C). Quite similar (if not identical) results were obtained when using the Hic-5 KD SKOV3 clone sh-S2 (data not shown). Lastly, Hic-5 suppression had a significant impact on SKOV3 cells sensitivity to cisplatin and paclitaxel, as Hic-5 KD cells showed much higher sensitivity to the two drugs compared to Ctrl cells (Cisplatin: sh-S1 *p* = 0.0012; sh-S2 *p* = 0.0061); (Taxol: sh-S1 *p* = 0.0033; sh-S2 *p* = 0.0084) (see Supplementary Figure 6D). Finally, our cell cytotoxicity data obtained with both (A2780s and SKOV3) cell lines were supported by data generated using the kmplotter tool, where lower expression of Hic-5 showed improved PFS survival (*p* = 0.028) in patients receiving both CT treatments (cisplatin and paclitaxel) (see Supplementary Figure 2D).

### Modulations of Hic-5 expression in EOC cells results in altered Hic-5 interaction with its partners/effectors, confirming its direct effect on EMT regulation

Consistent with its function as a molecular adaptor/scaffold protein, Hic-5 interacts with different binding partners and effectors under normal and pathological conditions, including cancer [44]. We decided to verify if alterations in Hic-5 expression in EOC cells could affect Hic-5 interactions with some members of its protein interactome.

TGFβ1-induced Hic-5 expression has been reported to reversibly correlate with its closest homolog – the focal adhesion protein paxillin, as it was suggested that these two proteins work cooperatively acting as molecular scaffolds to coordinate cellular attachment and migration [9, 14]. Among the different members of the paxillin family, the paxillin δ isoform shows high expression in cells of the epithelial phenotype, while as noted before, Hic-5 is specifically expressed in cells of mesenchymal origin [4]. Interestingly, when examining paxillin δ protein expression in the SKOV3 Ctrl and A2780s Ctrl cells, we observed an opposing pattern of expression, where SKOV3 shows very low paxillin δ expression, while A2780s shows a relatively high paxillin δ expression (Figure 5A and 5B). Moreover, TGFβ1 treatment of A2780s cells resulted in decreased paxillin δ protein expression (Figure 5B), and a similar effect was observed upon Hic-5 ectopic expression in A2780s cells (Supplementary Figure 1D), while some increase in paxillin δ protein expression was observed in the SKOV3 Hic-5 KD clone (Figure 5A). Collectively these data suggest that there might be a reciprocal functional association between these two close focal adhesion homologs in EOC cells, which could be directly modulated by Hic-5.

Another focal adhesion scaffold protein, the focal adhesion kinase (FAK) has been reported to coordinate certain remodeling complexes to maintain the mesenchymal phenotype of cells [45]. It is also suggested that Hic-5 may be in part modulating the process of EMT through a FAK dependent mechanism [8, 14], and that FAK phosphorylation actually plays an important role in EMT-related phenotypic changes [46–48]. Indeed, we found that the TGFβ1-induced Hic-5 expression in A2780s cells was associated with increased expression of the phosphorylated form of FAK (Figure 5B), as the ectopic expression of Hic-5 in these cells also induced a similar effect (Supplementary Figure 1D). Consistent with these findings, the knockdown of the Hic-5 gene in the mesenchymal cell line SKOV3 resulted in the suppression of the FAK phosphorylation form, which was not restored upon consecutive TGFβ1 treatment (Figure 5A).

**Figure 7:**
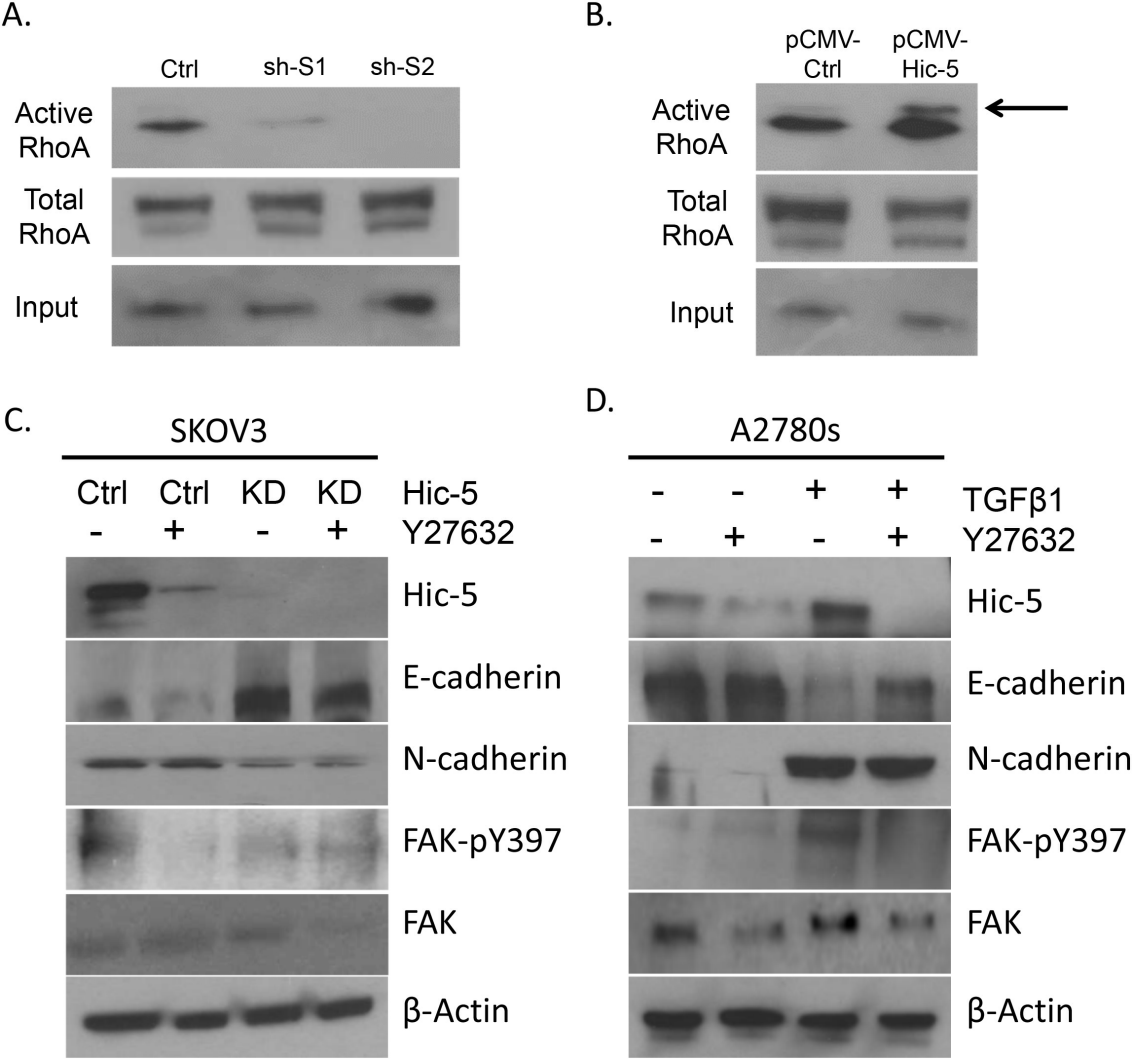
Assessment of RhoA activity and ROCK inhibition in EOC cells **(A)** GTP-Rhotekin RhoA pull-down samples from cell lysates of SKOV3 mock-transfected control (Ctrl) and shRNA-Hic-5 knockdown clones (KD) (sh-S1 and sh-S2) were immunoblotted with a Rho specific antibody for assessment of the active protein levels using Western blot. Cell lysates were also immunoblotted with a Rho specific antibody for total Rho protein levels. **(B)** GTP-Rhotekin RhoA pull-down samples from cell lysates of A2780s control clone (pCMV-Ctrl) and Hic-5 pCMV clone (pCMV-Hic-5) were immunoblotted with a Rho specific antibody for assessment of the active protein levels using Western Blot. Cell lysates were also immunoblotted with a Rho specific antibody for total Rho protein levels. **(C)** SKOV3 mock-transfected control (Ctrl) and shRNA-Hic-5 knockdown (KD) (sh-S1 and sh-S2) cells were either untreated (-) or treated (+) with 10mM of the ROCK inhibitor (Y27632) over a period of 48 hr. Western blot analysis were performed on cell lysates to examine protein expression of Hic-5, E-cadherin, N-cadherin, FAK-pY397, and FAK. **(D)** SKOV3 mock-transfected control (Ctrl) and shRNA-Hic-5 knockdown (KD) (sh-S1 and sh-S2) clones were either untreated (-) or treated (+) with 10mM of the ROCK inhibitor (Y27632) followed by stimulation without (-) or with (+) 5ng/ml of TGFβ1 for 48 hr. Western blot analysis were performed on cell lysates to examine protein expression of Hic-5, E-cadherin, N-cadherin, FAK-pY397, and FAK. β-actin was used as the loading control.

Additionally, Hic-5 seems to contribute to the EMT process through interactions with the RhoA/ROCK-dependent pathway [14, 17], also suggestive for a feed-forward interrelationship between Hic-5 and RhoA/ROCK [17]. We first verified if the expression of Hic-5 in our EOC cell lines does have a direct influence on RhoA activity. SKOV3 Hic-5 KD clones actually displayed suppressed RhoA activity, when measured using the GTP-Rhotekin RhoA pull-down assay (Figure 7A). Accordingly, we observed an increase in RhoA activity in A2780s cells ectopically expressing Hic-5 (the pCMV Hic-5 clone) compared to the pCMV-Ctrl; see Figure 7B).

Next we sought to examine a possible regulation by the kinase ROCK, as initially SKOV3 Hic-5 KD and Ctrl cells were treated with the ROCK inhibitor Y27632. ROCK inhibition reduced Hic-5 expression and inhibited FAK phosphorylation in the Ctrl cells (Figure 7C); however, EMT markers did not show any expression alterations upon treatment with the ROCK inhibitor, which supports previous reports indicating that the EMT marker E-cadherin maybe regulated independently of ROCK [14, 18], and which may also explain the absence of any change in N-cadherin expression upon treatment with the ROCK inhibitor (Figure 7C). Moreover, treating the A2780s cells with both TGFβ1 and the ROCK inhibitor showed that ROCK inhibition is sufficient to block the TGFβ1-mediated Hic-5 induction (Figure 7D), as ROCK inhibition similarly suppressed FAK phosphorylation in these cells (Figure 7D). Overall, our data are indicative that EMT modulation by Hic-5 in EOC cells maybe RhoA/ROCK dependent.

### Molecular mechanisms of Hic-5 action in EOC cells

To better understand the molecular mechanisms of Hic-5 action in EOC cells, we initially compared the gene expression of the selected shRNA-Hic-5 SKOV3 clones (sh-S1 and sh-S2) against the corresponding Ctrl clone. All microarray experiments were performed in duplicates, as two hybridizations were carried out for the Hic-5 KD clones against the corresponding Ctrl, using a fluorescent dye reversal (dye-swap) technique. For both comparisons, a subset of common differentially expressed genes was selected by initial filtering on confidence at *p*-value ≤ 0.05, followed by filtering on expression level (≥ 2 fold). Using these stringent selection criteria, we found 938 genes were upregulated and 1116 were downregulated in SKOV3 cells upon Hic-5 knockdown (see Supplementary Table 1).

**Figure 8:**
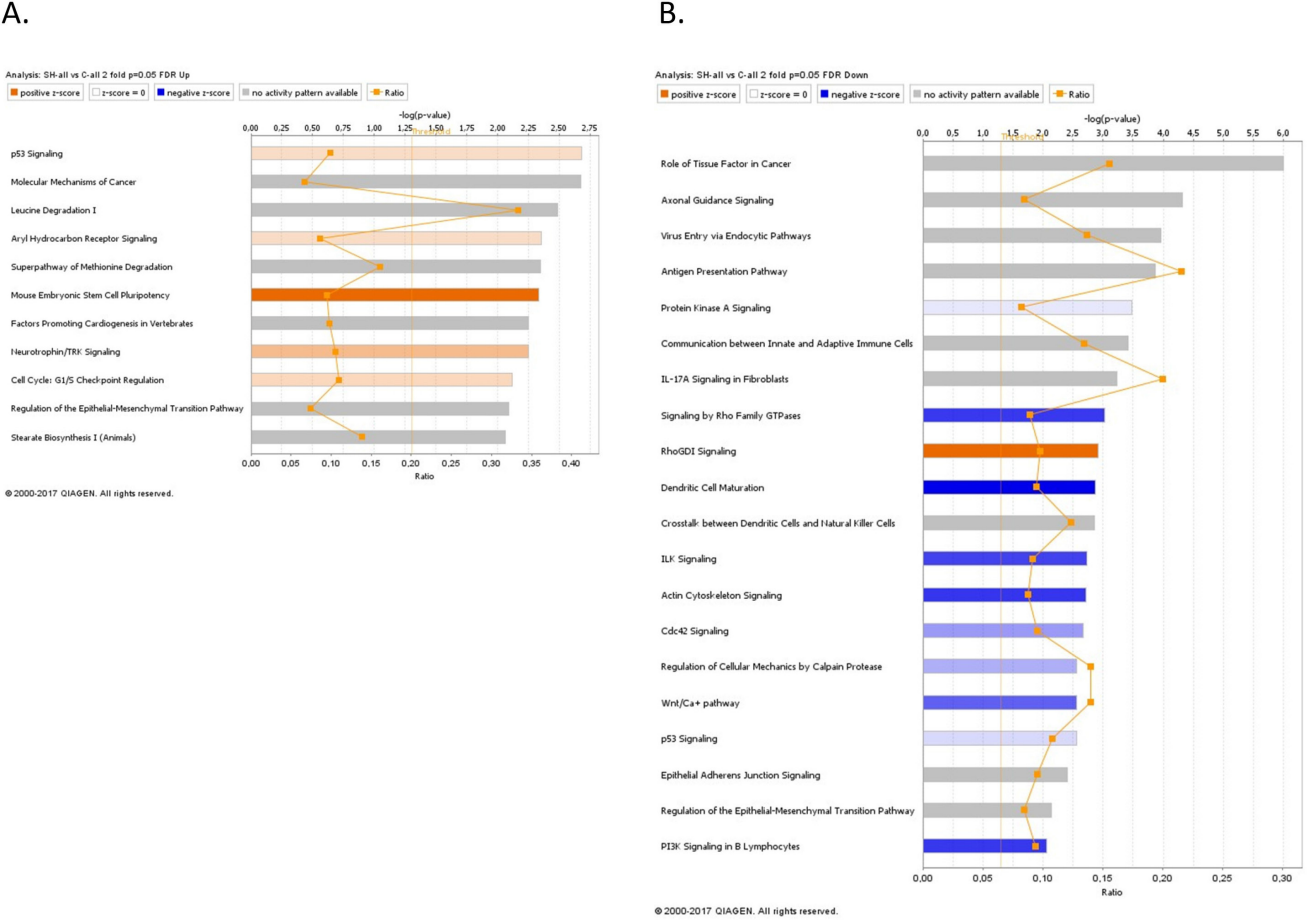
Canonical pathway analysis for a dataset of differentially expressed genes (≥ 2-fold) following Hic-5 suppression in SKOV3 cells **(A)** Canonical analysis of upregulated genes; **(B)** Canonical analysis of downregulated genes. Top functions that meet a Bonferroni-Holm multiple testing correction *p*-value of 0.05 are displayed.

**Figure 9:**
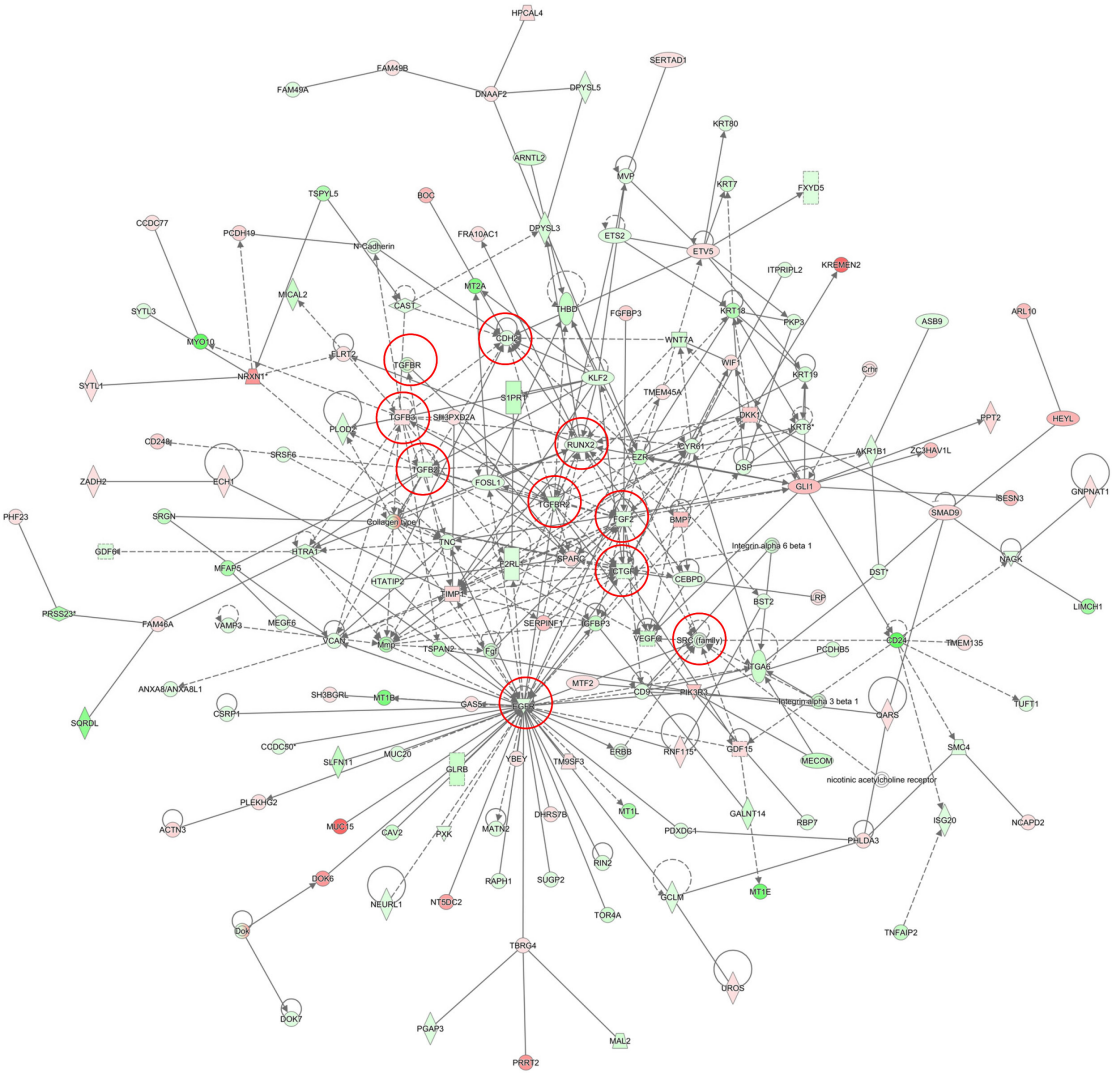
Network analysis of dynamic gene expression in SKOV3 cells based on the 2-fold gene expression list obtained following Hic-5 knockdown The five top-scoring networks of up- and down-regulated genes were merged and are displayed graphically as nodes (genes/gene products) and edges (the biological relationships between the nodes). Intensity of the node color indicates the degree of up- (red) or down-regulation (green). Nodes are displayed using various shapes that represent the functional class of the gene product (square, cytokine, vertical oval, transmembrane receptor, rectangle, nuclear receptor, diamond, enzyme, rhomboid, transporter, hexagon, translation factor, horizontal oval, transcription factor, circle, other). Edges are displayed with various labels that describe the nature of relationship between the nodes: __ binding only, → acts on. The length of an edge reflects the evidence supporting that node-to-node relationship, in that edges supported by article from literature are shorter. Dotted edges represent indirect interaction. Circled nodes in red represent the major gene nodes examined in this study.

The gene expression profiling experiments supported our *in vitro* observations of the major alterations associated with deregulations of the EMT pathway in the SKOV3 cells following Hic-5 suppression. Pathway and network analyses generated through the use of the Ingenuity Pathway Analysis (IPA) software were indicative for predominant upregulation of canonical pathways related to cell cycle G1/S checkpoint regulation and EMT regulation following Hic-5 suppression (Figure 8A), while canonical pathways downregulated upon Hic-5 KD were related to Protein Kinase A (PKA) signaling, IL-17A signaling, ILK signaling. Importantly, we also found downregulation of pathways related to signaling by Rho family GTPases, RhoGDI signaling, actin cytoskeleton signaling, epithelial adherens junction signaling and EMT (Figure 8B). Common networks obtained upon merging the top-scoring networks recognized some important gene nodes that were specifically up- or downregulated upon Hic-5 suppression in SKOV3 cells (Figure 9). Quite interestingly, genes and associated interaction partners that were downregulated upon Hic-5 KD comprised members and/or associated partners of the TGFβ pathway (including TGF-β2, TGFβR, TGFβRII, FGF2, CTGF, FGFR, SRC, RUNX2 and CDH2), that are commonly implicated in inducing EMT in cancer (Figure 9).

**Figure 10:**
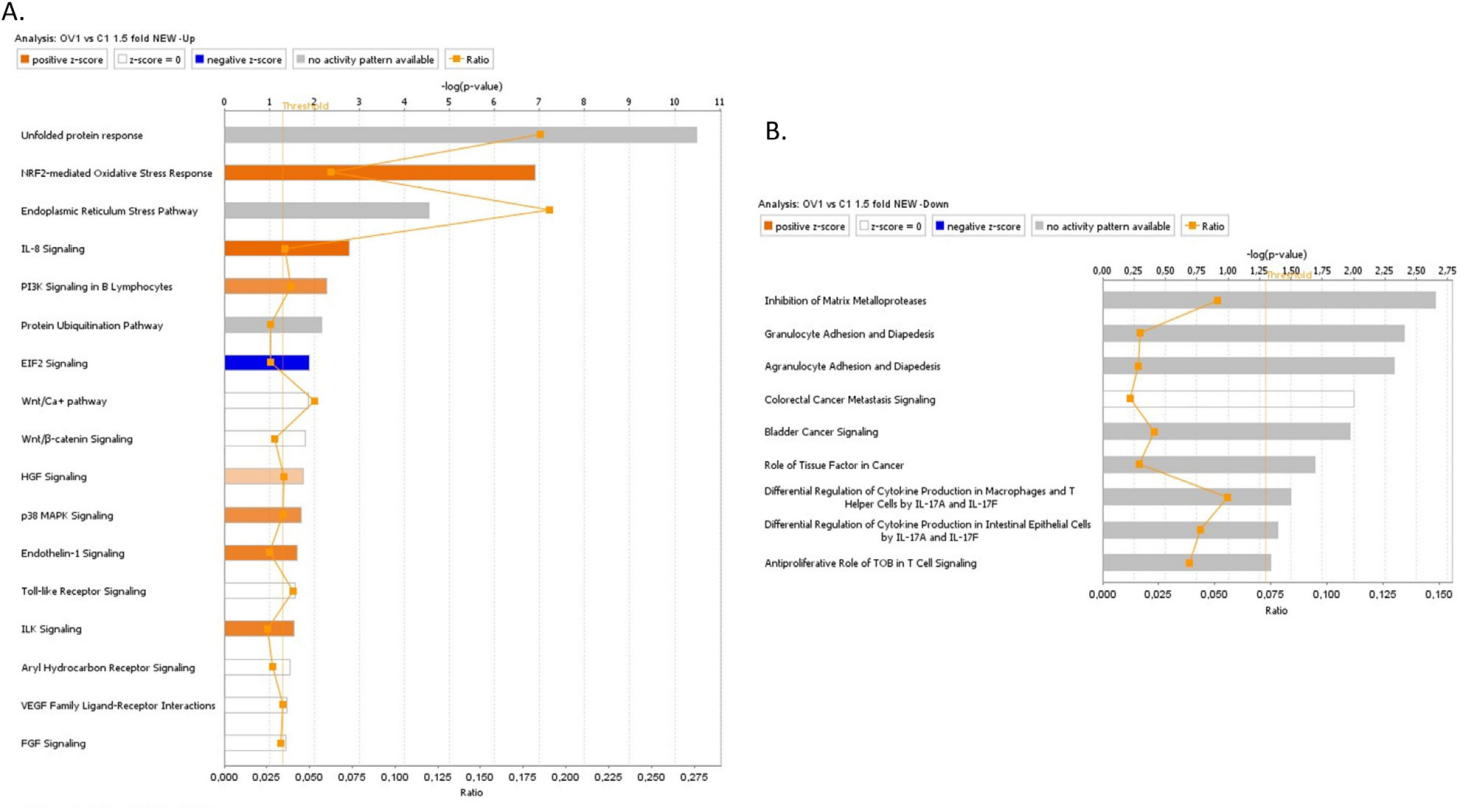
Canonical pathway analysis for a dataset of differentially expressed genes (≥ 1.5-fold) following ectopic expression of Hic-5 in the A2780s cells **(A)** Canonical analysis of upregulated genes; **(B)** Canonical analysis of downregulated genes. Top functions that meet a Bonferroni-Holm multiple testing correction *p*-value of 0.05 are displayed.

**Figure 11:**
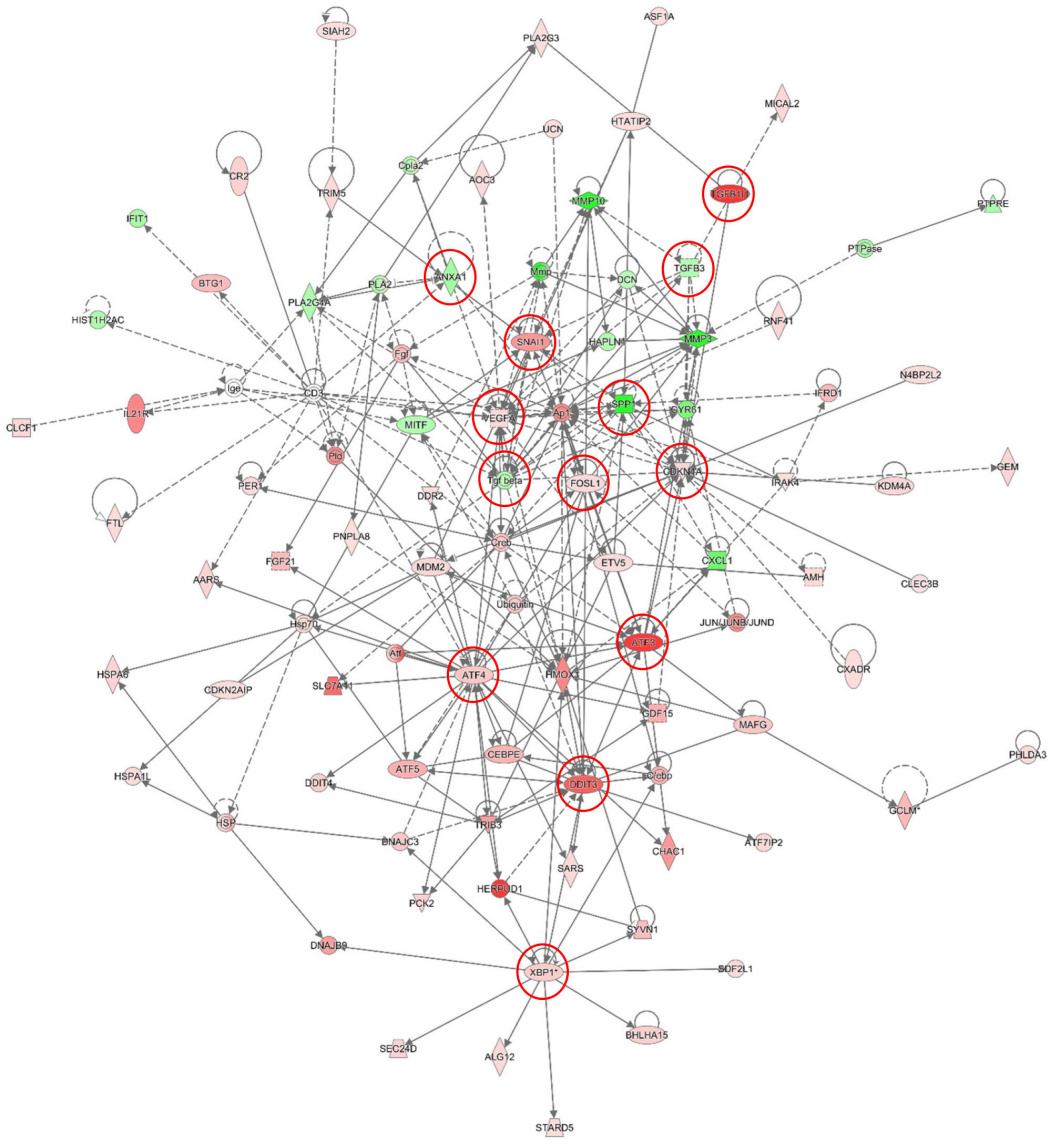
Network analysis of dynamic gene expression in A2780s cells based on the 1.5-fold gene expression list obtained following ectopic expression of the Hic-5 gene The five top-scoring networks of up- and down-regulated genes were merged and are displayed graphically as nodes (genes/gene products) and edges (the biological relationships between the nodes): see legend of Figure 9 for details.

Gene expression analysis were also performed on the A2780s cells ectopically expressing the Hic-5 gene (pCMV-Hic-5), and similarly, differentially expressed genes were selected by initial filtering on confidence at *p*-value ≤ 0.05, followed by filtering on expression level (≥ 1.5 fold). Using these selection criteria, we found 283 genes were upregulated and 47 were downregulated in the A2780s pCMV-Hic-5 clone compared to the Ctrl; (see Supplementary Table 2). Similarly, these gene expression data supported the Hic-5 implication in EMT regulation and possibly, EOC progression. Canonical pathway analysis showed that the upregulated pathways following ectopic expression of Hic-5 in A2780s cells were related to IL-8 signaling, PI3K signaling, EIF2 signaling, Wnt/Ca+ pathway, Wnt/β-catenin signaling, p38 MAPK signaling, and ILK signaling pathways, all involved in EMT induction in cancer (Figure 10A). Accordingly, downregulated pathways were functionally associated with inhibition of MMPs, differential regulation of IL-17, and finally a downregulation in the antiproliferative role of TOB in T cell signaling (Figure 10B). All these downregulated pathways are implicated in controlling the expression of the TGFβ3 gene and some MMP genes, again suggesting for a link between Hic-5 expression and EMT regulation. Network analysis was also indicative for the upregulation of a number of key gene nodes known to be involved in the regulation of the EMT pathway (including SNAI1, VEGFA, CDKN1A, FOSL1, ATF3, ATF4, DDIT3, XBP1), and finally the Hic-5 gene referred to as TGFB1I1 in the network were shown to be highly upregulated (Figure 11). Accordingly, some of the major downregulated gene nodes included: ANXA1, TGF beta complex, SPP1 and TGFB3 (Figure 11). To validate microarray results, we arbitrarily selected differentially expressed genes and quantified their expression by qPCR in both the SKOV3 and A2780s cells following knockdown and overexpression of the Hic-5 gene, and examined their mRNA expression compared to their Ctrls. Supplementary Figure 7 summarizes the gene expression measurements of all validated genes. We found that both methods (microarray analysis and qPCR) detected similar patterns for the up- and down-regulated genes selected for validation.

### *In vivo* examination of the effect of Hic-5 knockdown in tumor formation and survival in severe combined immunodeficient (SCID) mice

**Figure 12:**
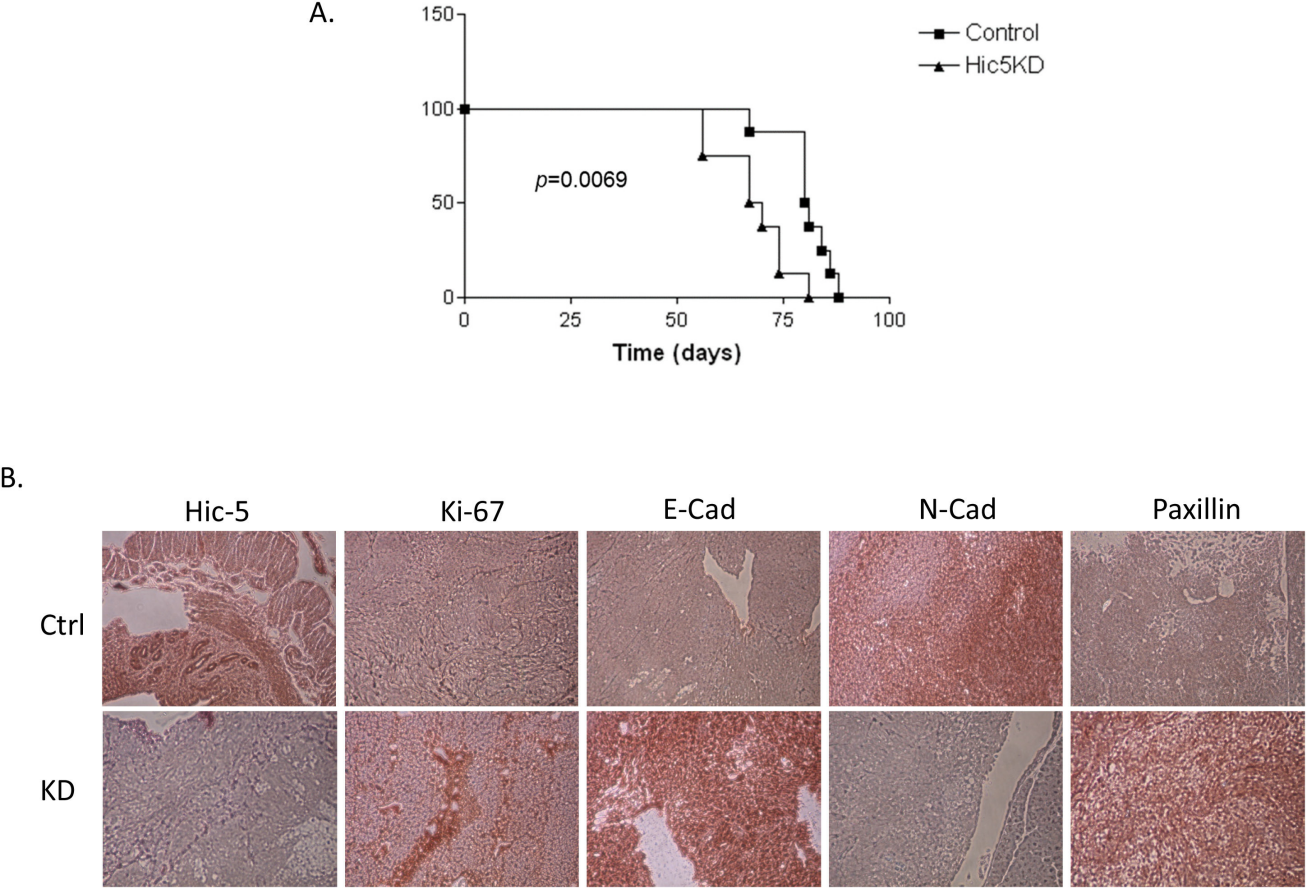
*In vivo* examination of the effect of Hic-5 knockdown in tumor formation and survival in immunodeficient (SCID) mice **(A)** Survival curves for mice injected with mock-transfected control (Ctrl), and shRNA-Hic-5 knockdown (KD) (sh-S1) SKOV3 cells. The median survival of mice injected with the Ctrl cells (81 days, n = 8). Survival of mice injected with the Hic-5 KD cells was significantly shorter than the Ctrl cells (68 days; *p =* 0.0069, Log rank test). **(B)** Representative IHC images of Hic-5, E-cadherin, N-cadherin, Paxillin δ, and Ki-67 expression in tumor tissues extracted from mice injected with the mock-transfected control (Ctrl), and shRNA-Hic-5 knockdown (KD) (sh-S1) SKOV3 cells.

Our *in vitro* observations suggest that morphological and functional changes upon Hic-5 KD in SKOV3 cells are associated with MET induction, and we thus wanted to explore if/how these changes affect the potential of EOC tumor growth and proliferation *in vivo*. EOC spreads by intraperitoneal (IP) sloughing, lymphatic invasion, and hematogenous dissemination [49]. IP dissemination is the most common; after malignant cells have evaded from the ovarian capsule, they are shed from the tumor surface into the peritoneal cavity where they follow normal routes of peritoneal fluid [50]. Hence, IP injection of cancer cells in animal models can accurately model advanced disease, as EOC metastases frequently appear disseminated throughout the peritoneum [51]. We used a similar *in vivo* approach; thus, Ctrl and shRNA-Hic-5 (clone sh-S1) SKOV3 cells were IP injected in SCID mice (n=8 per experimental group). Mice injected with Ctrl cells displayed a significantly longer survival (*p* = 0.0069) than those injected with Hic-5 KD cells, reaching endpoint on average 81 (+/- 2.24 SEM) days post and 68 (+/- 3.09 SEM) respectively (Figure 12A). Interestingly, the Hic-5 KD cell line resulted in a very different pathophysiology than that observed with mice injected with the Ctrl cells. The Hic-5 KD group of mice developed significantly greater (*p* < 0.001) average tumor mass (5.2g +/- 0.72g), representing an average tumor burden of 24% (Supplementary Figure 8A-8C). In comparison, mice injected with the Ctrl cells displayed an average tumor mass at endpoint of 2.5g +/-0.4, and their tumor burden was 11% (Supplementary Figure 8A-8C). There were no ascites present in any of the mice in the KD group, while ascites volumes of (1.69 +/- 0.72) were observed in the Ctrl group (Supplementary Figure 8D).

Consistent with our previous data *in vitro*, tumor specimens derived from sh-S1 cells showed very low staining intensity for the Hic-5 gene, while these tumors stained strongly for E-cadherin when compared with Ctrl SKOV3 derived tumors, whereas N-cadherin was only present following the injection of Ctrl cells (Figure 12B). These data sustain *in vivo* our findings for the implication of Hic-5 in controlling the EMT process. The expression of the proliferation marker Ki-67 was also induced in tumor tissues derived from mice injected with the Hic-5 KD cells (Figure 12B). Finally, relative induction of Paxillin expression following Hic-5 knockdown in SKOV3 cells was also confirmed *in vivo* by IHC (Figure 12B).

## DISCUSSION

The mechanisms for the tumorigenesis, progression and biological aggressiveness of EOC have not been yet fully clarified. Using an epigenomics technology, we have recently identified the Hic-5 gene as a novel potentially hypomethylated target in advanced EOC [7]. In the present study we demonstrate that Hic-5 is overexpressed in HG serous ovarian tumors as compared to normal ovarian tissues, suggestive for epigenetic regulation of Hic-5 during EOC etiology.

Hic-5 belongs to the LIM domain protein superfamily [52] and is also characterized as a focal adhesion adaptor protein that has the ability to shuttle between the cell membrane and the nucleus [19], allowing it to function as an adaptor protein, as well as a transcriptional receptor coactivator [53]. The functional implications of Hic-5 include its role in the morphological and phenotypic organization of cells, in addition to its role in cellular adhesion and motility [13, 14].

Different reports suggest for Hic-5 implications in malignant diseases, as Hic-5 has been intensively studied for its role in modulating the TGFβ signaling pathway and ECM remodeling during prostate cancerogenesis [10, 11, 17, 30, 36-40, 54-57]. Similarly, Hic-5-mediated regulatory functions were shown to induce metastasis in breast cancer [14, 32, 55], melanoma [33] and hepatocellular carcinoma [58].

In this study, we have examined for the first time the potential role of Hic-5 in ovarian tumorigenesis. Previous reports indicated that TGFβ1 induction is rather mandatory for the Hic-5-mediated regulation of EMT in epithelial cells [14-17, 19]. Our data confirmed that TGFβ1 treatment of epithelial-type EOC cell lines with low endogenous Hic-5 expression (A2780s and A2780cp) resulted in the upregulation of the Hic-5 protein and triggered EMT. However, we now present experimental evidence that Hic-5 can modulate EMT, and the EOC cellular phenotype independently of TGFβ1. Indeed, Hic-5 ectopic expression in A2780s alone was able to induce EMT-related phenotypic changes, similar to those obtained upon TGFβ1 treatment, and associated with functional cellular alterations (including enhanced cellular proliferation, migration, invasion and chemoresistance). Moreover, Hic-5 KD in the mesenchymal-type SKOV3 EOC cells triggered MET, related with decreased cellular proliferation, migration, invasion rates and augmented chemosensitivity. Importantly, consecutive TGFβ1 treatment of these epithelial-type Hic-5 KD SKOV3 cells did not induce any phenotypic (EMT-related) changes, suggesting that Hic-5 is required for the induction of EMT in EOC cells upon TGFβ1 treatment. These data suggest for a role of Hic-5 in maintaining the mesenchymal phenotype of EOC cells.

We also verified in EOC cells for interactions of Hic-5 with some of its previously characterized binding partners and effectors, and the impact of Hic-5 expression modulations on these interactions. Literature data were indicative for a relationship between Hic-5 and its closest homolog paxillin during EMT regulation [5]. The paxillin superfamily consists of 4 isoforms: paxillin α, β, γ and δ in addition to the closely related homologues (Hic-5 and Leupaxin) [18]. Interestingly, paxillin δ shows high expression in cells of the epithelial phenotype, while Hic-5 shows a specific expression in cells of mesenchymal origin [18, 30]. Similarly, we observed high paxillin δ protein expression in the epithelial-type EOC cells line A2780s, while almost no expression was observed in the mesenchymal-type EOC cell line SKOV3. Moreover, Hic-5 KD-mediated MET in SKOV3 cells also resulted in some induction in paxillin expression. Our data also confirm previous reports about Hic-5 interactions with FAK, as Hic-5 was shown to regulate FAK phosphorylation [24]. Indeed, Hic-5 KD in EOC cells resulted in the suppression of FAK phosphorylation, while Hic-5 ectopic expression in EOC cells led to FAK phosphorylation increase, in accordance with literature data indicative for the direct link between Hic-5 expression and FAK activity [56, 59, 60].

Additionally, it has been shown that Hic-5 interacts with members of the Rho GTPase family in the process of TGFβ1-mediated EMT induction [55]. The Rho GTPase family (and specifically Rac1, RhoA, and Cdc42) consists of well-established regulators of cell migration, coordinating adhesion dynamics and cytoskeleton remodeling [10, 61]. Rho GTPases were also shown to be involved in EOC metastasis by enhancing cell motility [11]. Recent reports have also demonstrated that Hic-5’s role in the process of EMT is RhoA and not Rac1 dependent [14, 33], and that RhoA activity drives cell invasion through the Rho kinase ROCK [31, 56]. ROCK plays an important role in the disassembly of cell-cell adherens junctions, and the regulation/development of the mesenchymal phenotype of cells [17]. Our results were indicative for a direct link between Hic-5 expression and RhoA activity in EOC cells that seems to be TGFβ1 independent, since Hic-5 KD resulted in reduced RhoA activity, while RhoA activity levels were accordingly induced upon Hic-5 overexpression. Moreover, ROCK inhibition suppressed the expression of Hic-5 in the mesenchymal-type SKOV3 cell line, and also resulted in blocking the TGFβ1-mediated induction of Hic-5 expression in the epithelial-type A2780s cell line. Overall, our data supports previous reports [9, 18] indicating that Hic-5’s role in the process of EMT is dependent on a RhoA/ROCK coordinated pathway, and suggests for a possible feedback mechanism for Hic-5 - RhoA/ROCK interactions in EOC cells.

Our *in vitro* experimental data were essentially confirmed upon analyzing global gene expression variations observed upon knockdown and/or overexpression of the Hic-5 gene in EOC cell lines. To our knowledge ours is the first study to evaluate differential gene expression following modulation of Hic-5 expression in cancer cells with both epithelial and mesenchymal phenotype. Indeed, the alterations in Hic-5 expression were indicative for a direct (TGFβ1-independent) modulation of multiple canonical pathways and gene nodes implicated in TGFβ signaling pathways and EMT. Thus, Hic-5 KD in SKOV3 cells affected multiple TGFβ pathway-related gene nodes (TGF-β2, TGFβR, TGFβRII), as well as important EMT-regulatory canonical pathways, including the PKA, the IL-17A and the ILK signaling pathways. As previously shown, a direct role of PKA interaction with TGFβ is required for EMT in pancreatic tumor cells [62, 63]. Moreover, a role for PKA in triggering EOC metastasis by regulating EOC cells migration and invasion has been also demonstrated [64]. Similarly, the IL-17A and the ILK signaling pathways were reported to be implicated in the process of EMT [65–69], in addition to their proven role in EOC metastasis [70–72]. We also observed a downregulation of canonical pathways related to the Rho family GTPases and RhoGDI signaling, which directly supports the observed suppression of RhoA GTPase activity upon Hic-5 KD in the SKOV3 cell line.

Accordingly, ectopic expression of Hic-5 in A2780s cells was associated with the upregulation of the IL-8, the EIF2, the Wnt/β-catenin and p38 MAPK canonical pathways, shown previously to be implicated in promoting malignancy through the regulation of EMT [73–78]. Interestingly, a decrease in the levels of phospho-p38 MAPK was observed upon Hic-5 suppression in breast cancer cells, confirming our findings suggestive for a role of Hic-5 in regulating p38 MAPK in cancer cells [15]. Hic-5 overexpression in A2780s resulted in the downregulation of the MMPs inhibition canonical pathway, shown previously to contribute to oncogenic EMT induction [79]. Moreover, some gene nodes linked to the TGFβ complex, previously suggested to be directly involved in EMT regulation and EOC dissemination [80–84], were found to be downregulated following Hic-5 induction.

Finally, our *in vivo* experiments suggest that mice IP injected with Hic-5 KD SKOV3 cells display lower survival rates, compared to mice injected with Ctrl cells. Interestingly, our data demonstrates a difference in the tumor formation capacity of the two cell types injected, as the injected Hic-5 KD SKOV3 cells led to increased tumor formation, whereas the Ctrl injected mice presented with ascites and a smaller tumor burden. We believe that the increased tumor growth observed with the Hic-5 KD cells might be primarily due to the inhibition of important signaling pathways of the EMT process, thus associated with a difference in the proliferation capacity of these cells undergoing MET. Our data are in accordance with a previous study showing that ectopic expression of Hic-5 in epithelial prostate cancer cells leads to significant suppression of tumor growth in immunodeficient mice, associated with restored sensitivity of the forming tumors to therapeutic castration [85]. Moreover, our data are in agreement with our and others previous findings, demonstrating that cancer cells with epithelial phenotype display reduced migration and invasion capacity *in vitro*, but exhibit superior capacity to colonize secondary sites *in vivo*, when compared to mesenchymal-like cancer cells [86–95]. Indeed, overexpression of different epithelial markers was observed in the metastatic sites of ovarian [96], breast [97–100], colorectal [101, 102], prostate [103, 104], lung [105, 106], and gastric [107] cancers, which is a strong indication that cancer cells in the primary tumor and their metastatic lesions share a similar epithelial nature.

In conclusion, we have shown that the focal adhesion molecule Hic-5 is significantly overexpressed in HG serous EOC tumors compared to LMP tumors and normal ovarian tissues, as epigenetic mechanisms might modulate Hic-5 overexpression in advanced disease. More importantly, we report that the Hic-5 gene can regulate the process of EMT in EOC cells in a TGFβ1 independent mechanism, suggesting for a role Hic-5 plays in maintaining the mesenchymal phenotype of EOC cells through possibly regulating/interacting with the RhoA/ROCK activation and signaling. Our *in vivo* data also support previous findings concerning the superior metastatic potential of cancer cells bearing epithelial phenotype as compared to cancer cells with mesenchymal-like morphology. Our study raises the need for a better understanding of the mechanisms by which focal adhesion adaptor molecules such Hic-5 can play in modulating tumor metastasis through the control of the process of EMT.

## MATERIALS AND METHODS

### Patients and tissue specimens

Snap frozen and formalin-fixed paraffin-embedded (FFPE) tissues of 117 EOC tumors were obtained at the Hotel-Dieu de Quebec Hospital, Quebec, Canada. These included 13 borderline, or LMP tumors and 104 HG adenocarcinomas. None of the patients received CT before surgery (see Table 1 for detailed clinicopathological characteristics). All tumors were histologically classified according to the criteria defined by the World Health Organization [108]. The CT treatment was completed for all patients and the response to treatment was known. Disease progression was evaluated following the guidelines of the Gynecology Cancer Intergroup [108]. Progression free survival (PFS) was defined as the time from surgery to the first observation of disease progression, recurrence or death. Thirteen normal ovarian samples were derived from women subjected to hysterectomy with oophorectomy due to non-ovarian pathologies. The study was approved by the Clinical Research Ethics Committee of the Hotel-Dieu de Quebec Hospital and all patients signed an informed consent for voluntary participation.

### Cell culture

The EOC cell lines OVCAR4, CaOV3 and SKOV3 were purchased from American Tissue Type Collection (Manassas, VA); OV90, OV2008, TOV112 and TOV21 cell lines were a kind gift from Dr. Anne-Marie Mes-Masson (Montreal University), while A2780s and A2780cp cell lines were a kind gift from Dr. Benjamin Tsang (Ottawa University). The two human ovarian surface epithelial (HOSE) cell lines; HOSE 6.3 and 17.1 were a kind gift from Dr. Francis Jacob (University Hospital Basel). The cell lines were passed in different culture media supplemented with 10% fetal bovine serum, as described previously [86, 109].

TGFβ1 was purchased from RandD systems and was used to induce EMT in EOC cells. EOC cells were treated with 5 ng/ml (SKOV3) or 10 ng/ml (A2780s and A2780cp) of TGFβ1 (R&D systems) for the indicated time periods. The ROCK (Y27632) inhibitor was purchased from MedChem express and cells were treated with 10 uM of Y27632 for the indicated time periods.

### Tissue microarrays (TMAs) construction and immunohistochemistry (IHC)

TMAs were constructed, as previously described [86, 110]. Briefly, one representative block of each ovarian tumor and normal ovarian tissue was selected for the preparation of the tissue arrays. Three 0.6 mm cores of tumor were taken from each tumor block and placed, 0.4 mm apart, on a recipient paraffin block using a commercial tissue arrayer (Beecher Instruments, Sun Prairie, WI). The cores were randomly placed on one of two recipient blocks to avoid IHC evaluation biases. Four micron thick sections were cut for the hematoxylin-eosin (HE) staining and IHC analyses.

IHC was performed, as previously described [86, 110]. Briefly, 4 μm tissue sections were deparaffinized and then heated in an autoclave for 12 min to retrieve the antigenicity before blocking with endogenous peroxidase. Following treatment with 3% H_2_O_2_ for 10 min to quench the endogenous peroxidise activity, sections were incubated with the appropriate primary antibody (see Supplementary Table 3 for a list of the antibodies used for IHC analysis). Sections were then incubated with a biotinylated secondary antibody (Dako, Carpinteria, CA) and then exposed to a streptavidin complex (Dako, Carpinteria, CA). Complete reaction was revealed by 3-3’ diaminobenzidine and slides were counterstained with hematoxylin. The protein expression was assessed by semiquantitative scoring of the intensity of staining and recorded as absent (0), weak (1+), moderate (2+) or strong (3+). The relationship between Hic-5 expression in serous ovarian carcinomas and normal ovarian tissues was evaluated by the Wilcoxon two-sample test. A significant association was considered when *p*-value was below 0.05. A Kaplan-Meier curve and the log-rank test were performed based on PFS values to test the effect of the intensity of Hic-5 (3, 2 versus 0, 1) on disease progression.

### Ectopic Hic-5 expression in A2780s cells

Hic-5 ectopic expression was performed as previously described [109]. Briefly, cDNA of the human Hic-5 gene (Myc-DDK-tagged) cloned in the pCMV6 entry eukaryotic expression vector (pCMV-Hic-5) was purchased from OriGene Technologies, Inc. (Rockville, MD). Transfection with ExGen 500 (Fermentas Canada Inc., Burlington ON) was carried out according to the manufacturer’s guidelines. Briefly, 1x10^5^ A2780s cells were plated onto 6x30-mm well plates and allowed to grow to 70% confluence. Ten microliters of ExGen 500 were added to 2 μg of plasmid DNA dissolved in 190 μl of 150 mM NaCl. The complexes were incubated at room temperature for 10 min and then overlaid onto the cells in 1.8 ml medium. The plates were then incubated at 37°C, 5% CO_2_ for 48 hr. Stably transfected clones were selected by adding neomycin (500 μg/ml) and were further cultivated for about 2 weeks. Cells were also mock-transfected with the empty pCMV6 vector, and stably transfected clones were isolated as controls. Western blot analyses of the Hic-5 and the DDK protein expression levels were performed for validation of Hic-5 overexpression of the selected Ctrl and Hic-5-overexpressing clones.

### Short hairpin RNA (shRNA) – mediated Hic-5 knockdown in EOC cells

The shRNA-mediated Hic-5 knockdown in EOC cells was done, as previously described [86]. Briefly, two Hic-5 shRNAs cloned into the pLKO.1-puro vector (targeting the Hic-5 mRNA sequences 5′-CAGTTCAACATCACAGATGAA-3′ and 5′-CGGTTGCTTCAGGAACTTAAT-3′) were retrieved from the Sigma Mission TRC human 1.5 shRNA library (clone numbers TRCN0000020100 and TRCN0000281350). Viral supernatants were generated by transfecting 293T cells with the shRNA constructs and the packaging vectors psPAX2 and pMD2.G (Addgene, Cambridge, MA). The high-titer lentiviral supernatants in the presence of 8 mg/ml polybrene were used to infect SKOV3 and TOV112 cells. Two days later, infected cells were treated with puromycin (0.5 mg/ml for SKOV3 and TOV112 cell lines) for the selection of stably-transduced clones. The pLKO.1-puro vector encoding a scramble sequence not matching any mammalian sequence was used for the generation of mock-transduced (Ctrl) clones. Stable clones with inhibited Hic-5 expression were evaluated and validated by quantitative RT-PCR, semi-quantitative PCR and Western blot.

### Functional assays

Cell proliferation was evaluated by Malassez cell counting as previously described [109]. In addition, cell proliferation (cell index) was checked by the xCELLigence Real-Time Cell Analyzer (RTCA) instrument, as previously described [86]. Colony formation assay was performed as previously described [110]. Wound healing assay was performed on the SKOV3 Hic-5 KD and Ctrl clones, cells were seeded into 10cm plates to 80–90% confluence and the cell monolayer was scratched in a straight line with a 200 μl pipette tip to create a “scratch”. Debris was removed with PBS and then the culture was re-fed with fresh medium. Images were taken at 0, 12 and 24 hr after the scratch to calculate the cell migration rate. Cell migration and invasion assays were performed as previously described [86, 109, 110]. Cell cycle flow cytometry analysis was performed as previously described [109], while the cell cycle phase distribution was calculated from the resultant DNA using the FlowJo software (v10). All statistical data were determined by a Student’s *t*-test, where *p* < 0.05 was considered significant.

### MTS cell cytotoxicity assay

A CellTiter 96 One Solution cell proliferation assay (MTS assay) was used as previously described [86] according to the manufacturer’s instructions (Promega). Cells were seeded (at 2 × 10^4^ cells/ml) on 96-well plates in triplicates and incubated for 72 hr with the different drugs used with concentrations ranging between 0.01 μM to 100 μM. After 72 hr, 20 μl of the CellTiter 96 One Solution from the kit was added to the culture medium containing the samples. The plates are then incubated for 1 to 4 hr at 37°C in a humidified, 5% CO_2_ incubator. The absorbance values (490 nm) were recorded with a plate reader and the relative cell numbers were then calculated.

### Semi-quantitative RT-PCR (sqRT-PCR)

SqRT-PCR was performed as previously described [110]. The GUSB gene was used as an internal standard. Primers were designed for these loci with the sequences freely available from the Entrez Nucleotide database and the Primer3 algorithm for primer design (http://www-genome.wi.mit.edu/cgi-bin/primer/primer3_www.cgi) (see Supplementary Table 4 for RT-PCR primer description).

### Quantitative PCR (qPCR)

Quantitative PCR was performed as previously described [86]. Briefly, total RNA was extracted by RNeasy Plus Mini Kit (QIAGEN) and cDNA was obtained by qScript™ cDNA SuperMix (Quanta BioSciences, Inc.). Primers were designed for these loci with the sequences freely available from the Entrez Nucleotide database and the Primer3 algorithm for primer design (http://www-genome.wi.mit.edu/cgi-bin/primer/primer3_www.cgi). The primers used for qPCR validation are listed in Supplementary Table 4. PerfeCTa® SYBR® Green FastMix® (Quanta BioSciences, Inc.) was used according to manufacturer’s instructions. PCR reactions were performed on Rotor-Gene RG-3000 Real Time PCR System (Qiagen), with 18S ribosomal RNA used as endogenous control. PCR volume was 20 μl, and conditions were as follow: initial cycle 50°C, 2 min, 95°C, 15 min; 45 cycles at 95°C, 20 s, 60°C, 20 s and 72°C, 20 s; final cycle 72°C, 30 s. Data were analyzed by the Rotor-Gene software using the comparative ΔΔCt method. The relative copy number was calculated based on the target gene/18S RNA ratio.

### Western blotting

Western blot analyses were performed as previously described [86, 109]. Briefly, protein lysates were prepared by resuspending cell pellets in Laemmli sample buffer containing 5% β-mercaptoethanol. Protein lysates were separated by 6 to 12% Tris-glycine gel electrophoresis and transferred onto a polyvinylidene difluoride membrane. The membranes were blocked with 4% non-fat dry milk in TBST (20 mmol/L Tris-HCl, 0.5 M NaCl, and 0.1% Tween 20), incubated with the appropriate primary antibody at 4°C overnight (Refer to Supplementary Table 3, for a list of the primary antibodies used in the study). After 3 × 15 min washes with TBST at room temperature, the membranes with the corresponding secondary antibody in TBST containing 4% non-fat dry milk for 1-2 hr at room temperature. Upon washing, the signal was visualized using ECL solution (Thermo Fisher Scientific, Waltham, MA) and detected on blue sensitive autoradiography film (Marsh Bio Products, Rochester, NY).

### RhoA activity assay

RhoA activity was monitored using the RhoA activation assay kit (Abcam) according to the manufacturer guidelines. Briefly, cells grown up to 80 – 90% confluence were washed twice with ice-cold PBS and resuspended in assay buffer provided. Upon incubation on on ice for 10 – 20 mins, cells were then detached by scraping with a cell scraper and cell lysates were centrifuged for 10 min at 4°C at 14,000 × g to remove any insoluble material. Forty μL of resuspended Rhotekin RBD agarose bead slurry was added to 1 mL of the cell lysate and the mix was then incubated at 4°C for 1 hr with gentle agitation. Beads were then pelleted by centrifugation for 10 seconds at 14,000 × g. and resuspended in 40 μL of 2X reducing SDS-PAGE sample. Subsequently, the precipitated GTP-Rho was detected by Western blot analysis using an anti-RhoA specific monoclonal antibody.

### Methylation-specific PCR (MSP)

Genomic DNA from the shRNA-Hic-5 KD clones sh-S1 and sh-S2 and the Ctrl was isolated using the DNeasy Blood and Tissue Kit (Qiagen, Canada). Bisulfite modification of genomic DNAs was done using the MethylDetector kit (Active Motif, Carlsbad, CA). MSP primer selection was performed using the Methyl Primer Express Software v1.0 (Applied Biosystems). PCR was done as for 30 cycles (94°C, 30 s; 94°C, 45 s, 60°C, 45 s, 72°C, 45 s; 72°C, 10 min). The band expanded with methylation-specific PCR primers corresponding to the DNA methylation in the promoter region was marked as “M”. The band expanded with non-methylation-specific primers was marked as “U”.

### Gene expression profiling and data analysis

Gene expression analysis was carried out as previously described [86]. Briefly, total RNA was extracted from the shRNA-Hic-5 KD clones (sh-S1, sh-S2), the pCMV-DDK transfected clone (pCMV-Hic-5) and their corresponding controls. The quality of the RNA samples was examined by capillary electrophoresis using the Agilent 2100 Bioanalyzer (Agilent). Fluorescently labeled cRNA targets were generated from 0.5 μg of total RNA from each corresponding cell line clone, using the Fluorescent Linear Amplification Kit (Agilent) and 10 mM Cyanine 3- or 5-labeled CTP (PerkinElmer), following the user’s manual. Cyanine labeled cRNA from the Hic-5-KD SKOV3 clones (sh-S1 and sh-S2) and the clone ectopically expressing Hic-5 (pCMV-Hic-5) were mixed with the same amount of reverse-color cyanine-labeled cRNA from their corresponding controls and hybridized on the Agilent Whole Human Genome microarrays, containing 44,000 genes. Array hybridization, washing, scanning, data extraction and analyses were performed as previously described [86]. Network analysis of the microarray data was completed using the Ingenuity Pathway Analysis (IPA) software (see http://www.Ingenuity.com). The microarray data have been deposited to the GEO database (http://www.ncbi.nlm.nih.gov/geo/) with accession number GSE98737.

### Peritoneal tumor formation in mice

Control SKOV3 cells, as well as cells from the SKOV3 shRNA-Hic-5 KD (sh-S1) clone (1 × 10^7^ cells in 500 μL of PBS), were IP injected into 8 × 8 week old CB17 SCID female mice (CB17/Icr-Prkdcscid/IcrIcoCrl strain code 236, Charles River) using a 25G5/8 needle, as previously described [86]. Mice were monitored daily by staff blinded to the cell type injected and euthanized when they reached a loss of wellness endpoint that was most often respiratory distress associated with ascites accumulation. The animals had free access to food and water and experiments were done in accordance with the Canadian Council on Animal Care's Guidelines for the Care and Use of Animals. Protocols were approved by the University of Ottawa Animal Care Committee.

## SUPPLEMENTARY MATERIALS TABLES








