# Supplementary material for: Hic-5 regulates epithelial to mesenchymal transition in ovarian cancer cells in a TGFβ1-independent manner
Source: Oncotarget. 2017 Jul 31;8(47):82506–30. doi: 10.18632/oncotarget.19714 (PMC5669907; doi:10.18632/oncotarget.19714)
Supplement: Supplementary file 1 [file oncotarget-08-82506-s001.pdf]

Hic-5 regulates epithelial to mesenchymal transition in ovarian cancer cells in a TGFβ1-independent manner

SUPPLEMENTARY MATERIALS

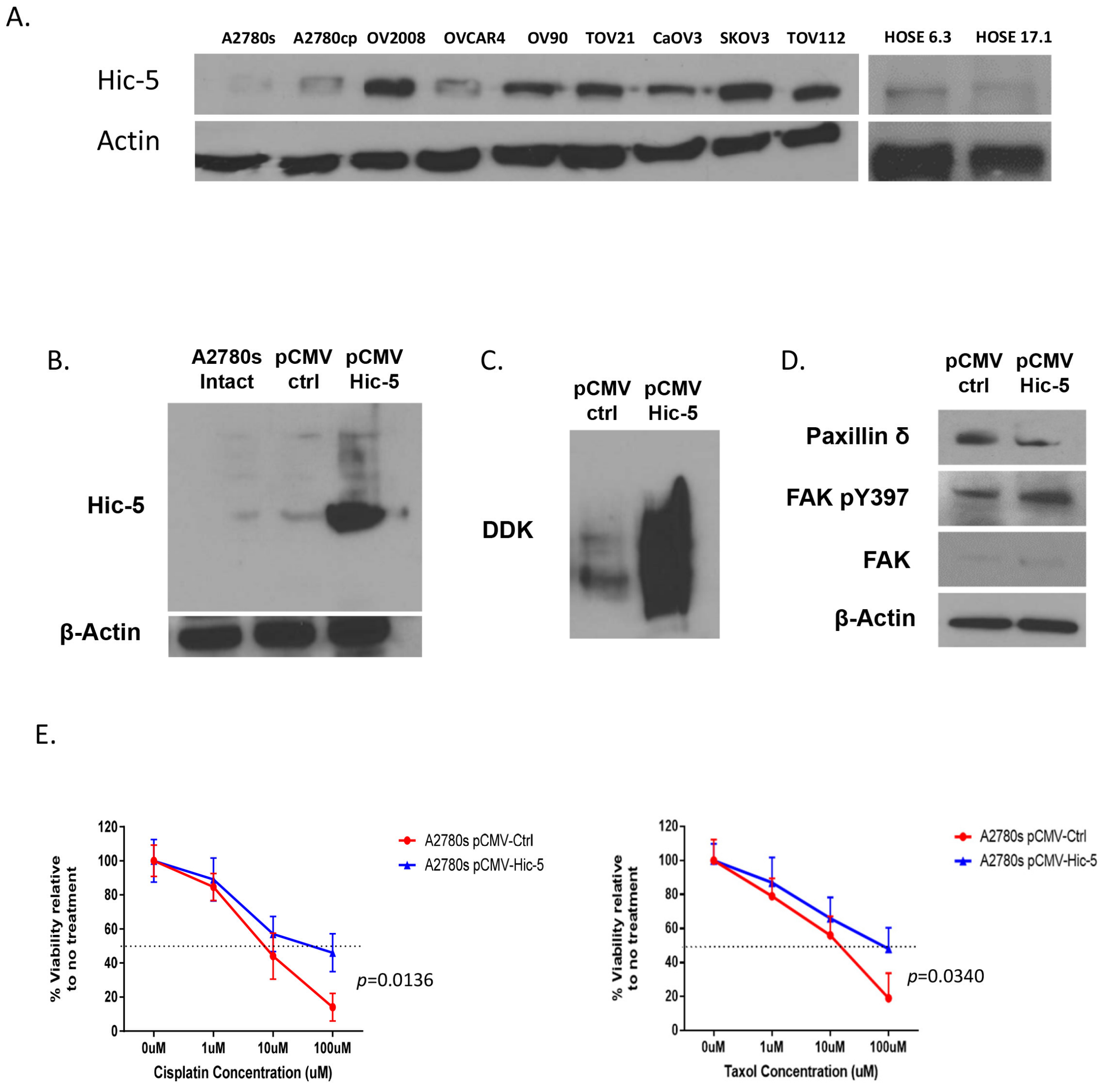

Supplementary Figure 1: (A) Western blot analysis of endogenous Hic-5 protein expression in different EOC cell lines, in addition to the two HOSE cell lines (HOSE 6.3 and HOSE 17.1). **(B)** Western blot confirmation of the ectopic expression of Hic-5 pCMV clone in A2780s cells (pCMV-Hic-5). Protein overexpression of Hic-5 was compared to A2780s intact cells and to the control clone (pCMV-Ctrl). **(C)** Western blot analysis of DDK expression (DYKDDDDK epitope) was performed for validation of Hic-5 overexpression in the A2780s cells. **(D)** Protein expression analysis of paxillin δ, FAK pY397, and FAK in control clone (pCMV-Ctrl) and Hic-5 pCMV (pCMV-Hic-5) A2780s cells. β-actin was used as a loading control. **(E)** Dose-response cytotoxicity curves upon cisplatin (left) and paclitaxel (Taxol) (right) treatment of A2780s cells. Treatment responses of the Hic-5 pCMV (pCMV-Hic-5) clone was compared to the control (pCMV-Ctrl) clone. Differences between A2780s pCMV-ctrl and A2780s pCMV-Hic-5 cells were determined by a Student's t-test; error bars denote mean ± SEM; ($p < 0.05$).

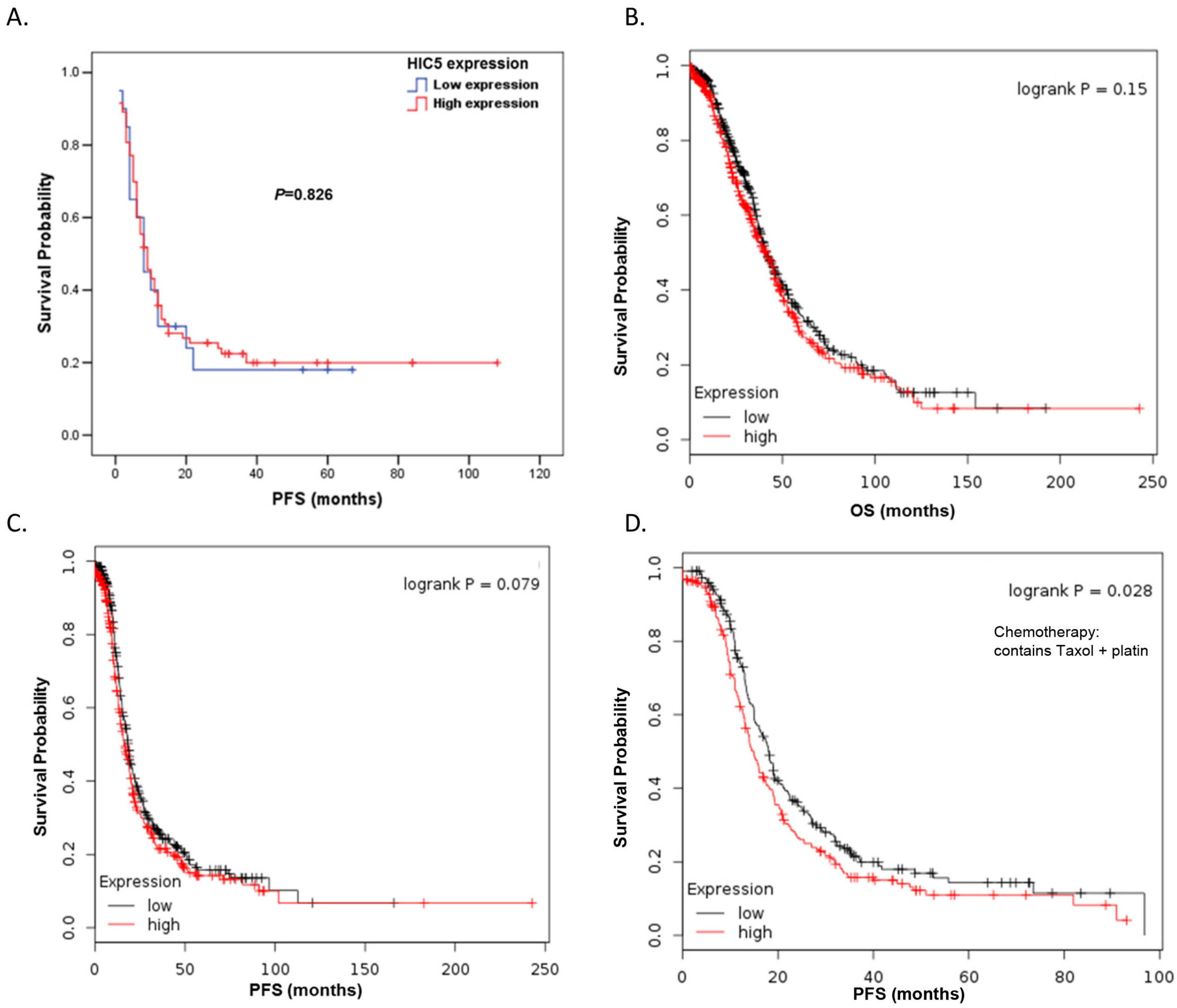

Supplementary Figure 2: (A) Kaplan-Meier curve for progression free survival (PFS) according to the level of Hic-5 IHC intensity in tumor samples of 103 serous EOC patients. **(B)** Relationship between Hic-5 expression and overall survival (OS) in ovarian cancer cohort (1287 EOC patients), using the kmplotter tool. **(C)** Relationship between Hic-5 expression and PFS in ovarian cancer cohort (1287 EOC patients), using the kmplotter tool. **(D)** Relationship between Hic-5 expression and PFS in ovarian cancer cohort (1287 EOC patients), restricting the analysis for patients treated with both CT conventional drugs (Taxol + platin). In all kmplotter analyses, OS and PFS were assessed in high-grade (Grade 3) serous EOC cases using the Jetprobe set for Hic-5 and a cutpoint at median.

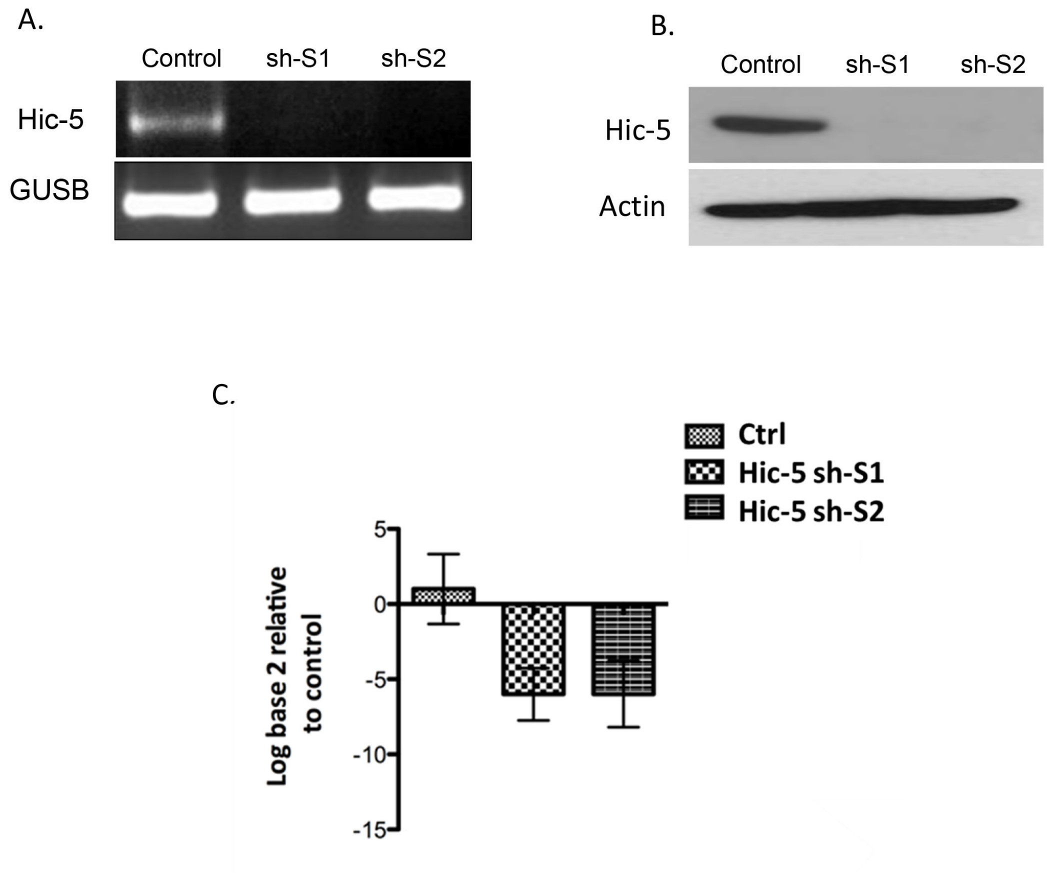

Supplementary Figure 3: **(A)** Semi-quantitative PCR (RT-PCR) analysis of Hic-5 mRNA expression levels in the shRNA-Hic-5 SKOV3 knockdown (KD) clones (sh-S1 and sh-S2), compared to the control clone (Ctrl). **(B)** Western blot analysis of Hic-5 protein expression in the shRNA-Hic-5 SKOV3 Knockdown (KD) clones (sh-S1 and sh-S2), compared to the mock-transfected control clone (Ctrl). β-actin was used as a loading control. **(C)** Quantitative PCR (qPCR) analysis of Hic-5 mRNA expression levels in the shRNA-Hic-5 SKOV3 knockdown (KD) clones (sh-S1 and sh-S2), compared to the mock-transfected control clone (Ctrl).

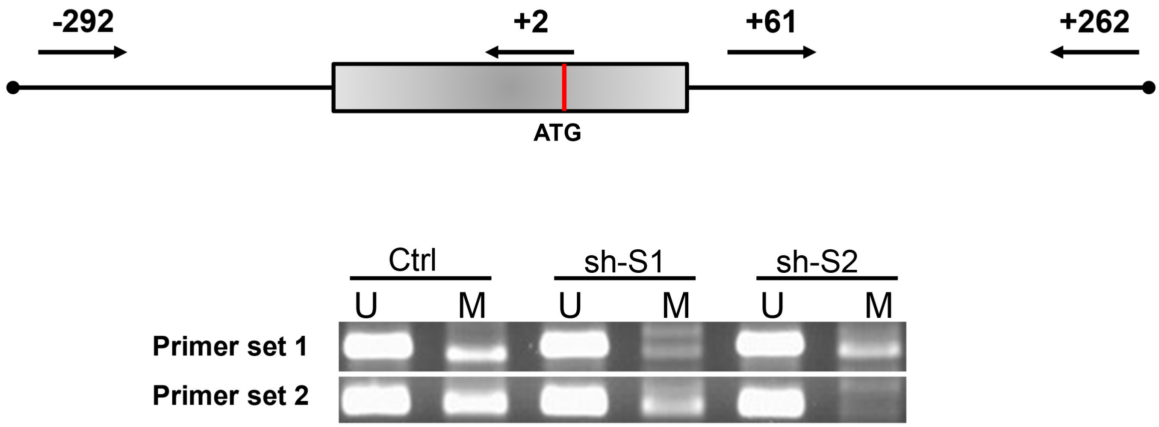

Supplementary Figure 4: Methylation-specific PCR (MSP) analysis of E-cadherin methylation in the control and Hic-5 sh-RNA knockdown (sh-S1 and sh-S2) clones. The indicated positions represent the number of nucleotides stretching up- or down-stream of the start (ATG) codon of the E-cadhedrin gene and covering its putative promoter region. The band expanded with methylation-specific PCR primers corresponding to the DNA methylation in the promoter region was marked as “M”. The band expanded with non-methylation-specific primers was marked as “U”.

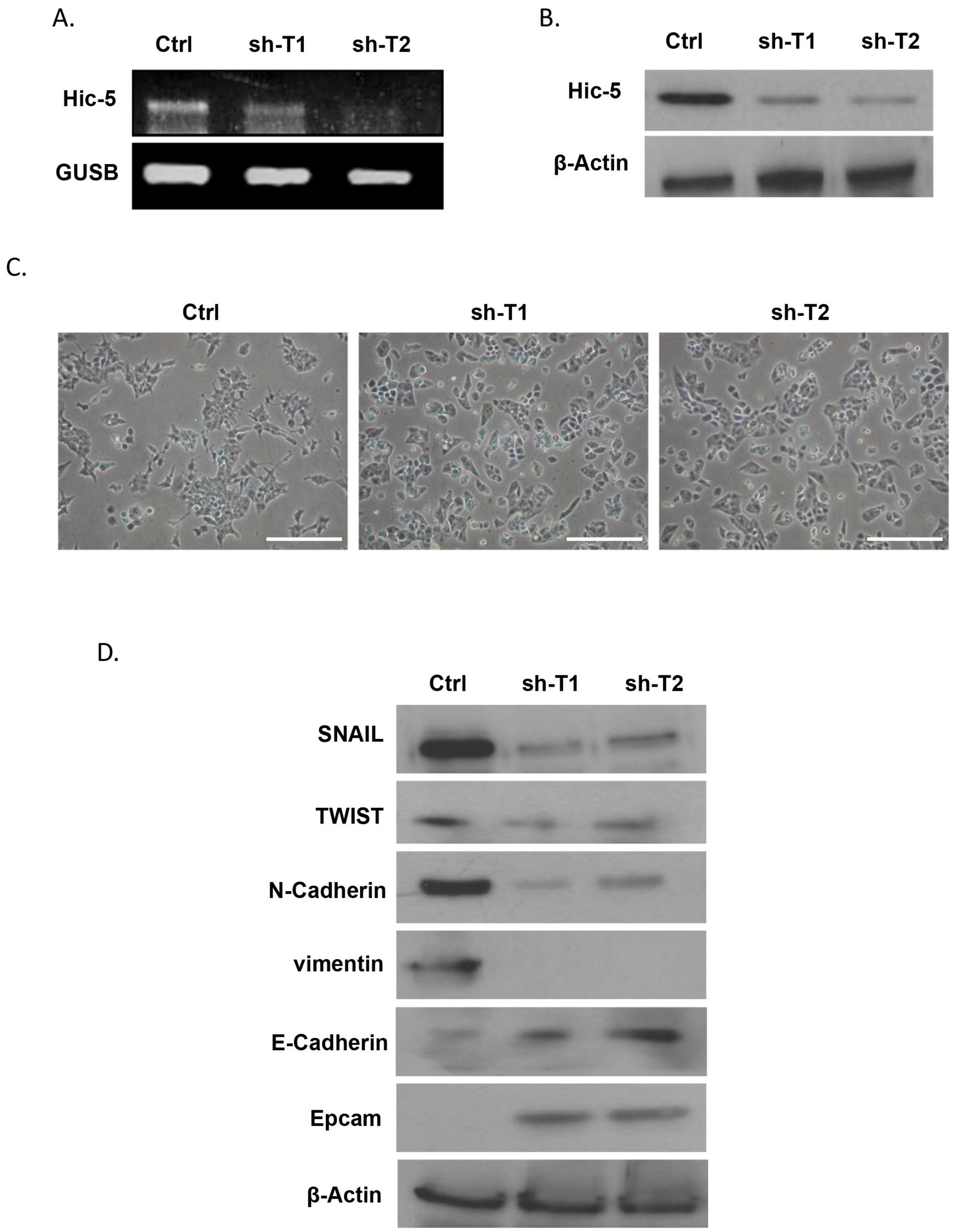

Supplementary Figure 5: (A) Semi-quantitative PCR (RT-PCR) analysis of Hic-5 mRNA expression levels in the shRNA-Hic-5 TOV112 knockdown (KD) clones (sh-T1 and sh-T2), compared to the mock-transfected control clone (Ctrl). **(B)** Western blot analysis of Hic-5 protein expression in the shRNA-Hic-5 TOV112 knockdown (KD) clones (sh-T1 and sh-T2), compared to the mock-transfected control clone (Ctrl). β-actin was used as a loading control. **(C)** Representative phase-contrast images of TOV112 mock-transfected control and shRNA-Hic-5 knockdown (KD) clones (sh-T1 and sh-T2). Scale Bar = 200 µm. **(D)** Western blot analysis of the expression of different EMT (epithelial and mesenchymal) markers in the control and the shRNA-Hic-5 knockdown (KD) TOV112 clones (sh-T1 and sh-T2) compared to the mock-transfected control clone (Ctrl) β-actin was used as a loading control.

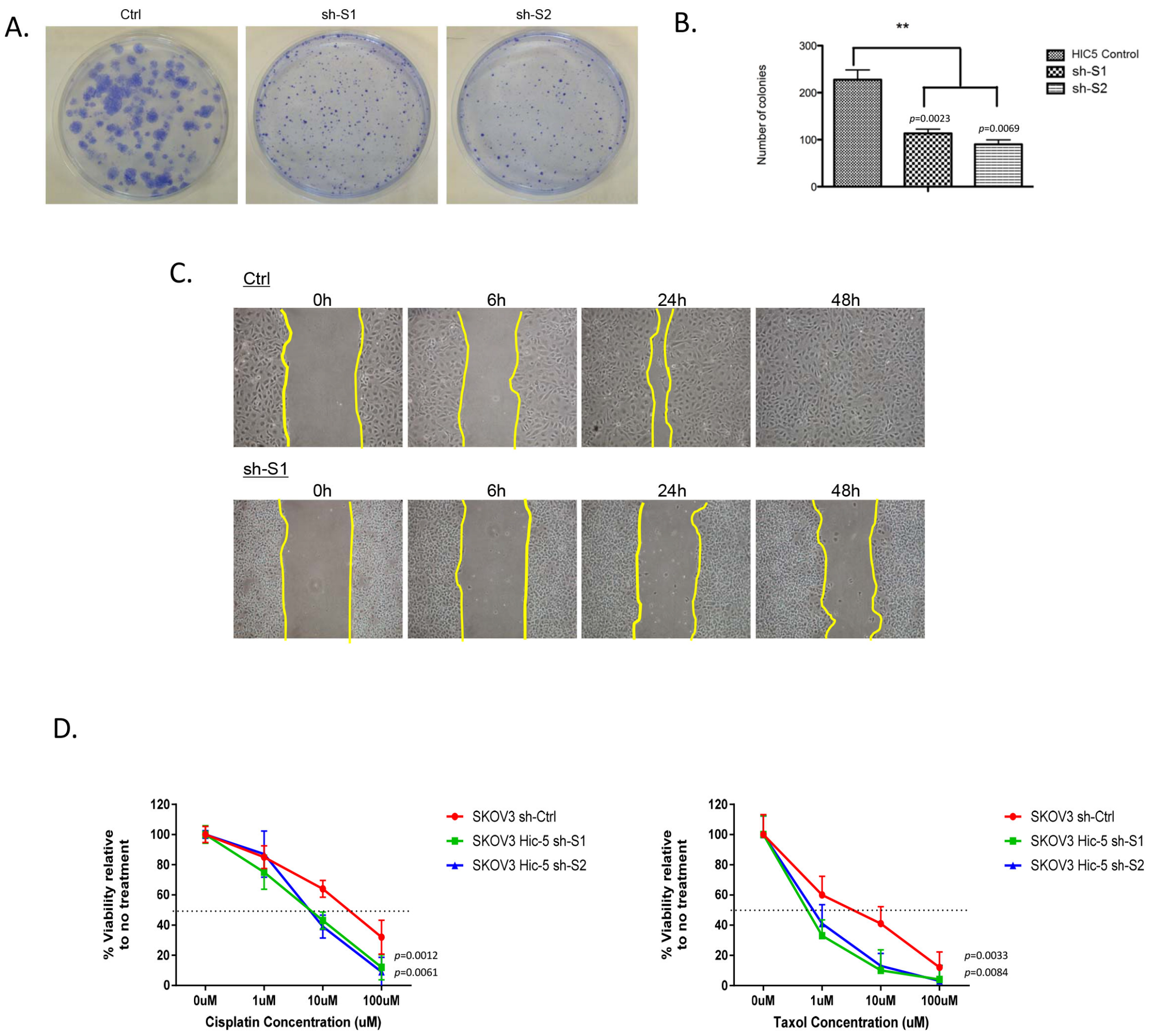

Supplementary Figure 6: (A) Representative images of colony forming assays following shRNA-Hic-5 knockdown (KD) in the SKOV3 cell line (sh-S1 and sh-S2). **(B)** Representative quantitative determinations of colony formation assay obtained by determining the colony area which focuses on adjusting for the percentage of the area covered by crystal violet stained cell colonies, and the intensity of the staining of the colonies per plate. Results are expressed as number of colony differences between shRNA-Hic-5 knockdown (KD) (sh-S1 and sh-S2) clones compared to the mock-transfected control clone (Ctrl). Differences in colony numbers were determined by a Student's t-test; error bars denote mean ± SEM; *indicates statistical significance ($p < 0.05$). **(C)** Following Hic-5 knockdown in the SKOV3 cell line, the cells were scratch-wounded with a micropipette tip (200 µl). Yellow lines indicate the wound borders at the beginning of the assay and were recorded at 0, 6, 24 and 48 h post-scratching. **(D)** Dose-response cytotoxicity curves upon cisplatin (left) and paclitaxel (Taxol) (right) treatment of SKOV3 cells following shRNA-mediated Hic-5 knockdown. Treatment responses of the shRNA- Hic-5 knockdown (KD) clones (sh-S1 and sh-S2) were compared to the mock-transfected control clone (Ctrl). Differences between SKOV3 Hic-5 Ctrl and Hic-5 sh-S1 and sh-S2 cells were determined by a Student's t-test; error bars denote mean ± SEM; ($p < 0.05$).

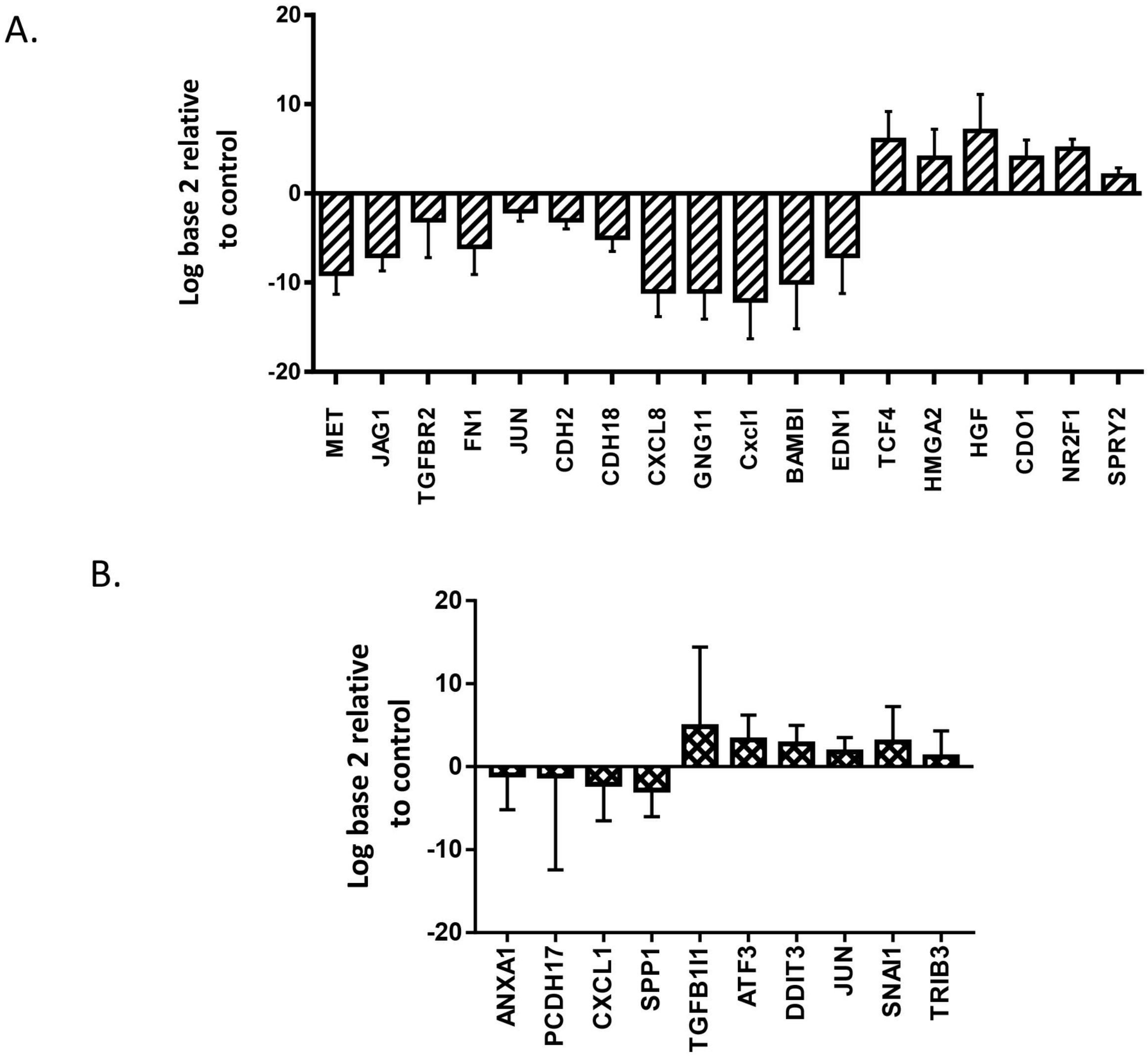

Supplementary Figure 7: (A) The figure shows bar graphs presentation of the differential expression of the selected genes in SKOV3 cells following Hic-5 knockdown compared to mock-transfected control SKOV3 cells. **(B)** The figure shows bar graphs presentation of the differential expression of the selected genes in A2780s cells following Hic-5 overexpression compared to mock-transfected control A2780s cells. The relative copy number was calculated based on the target gene/18S ribosomal RNA ratio. Values more than or equal to 1 represent gene upregulation and less than 1 display gene downregulation.

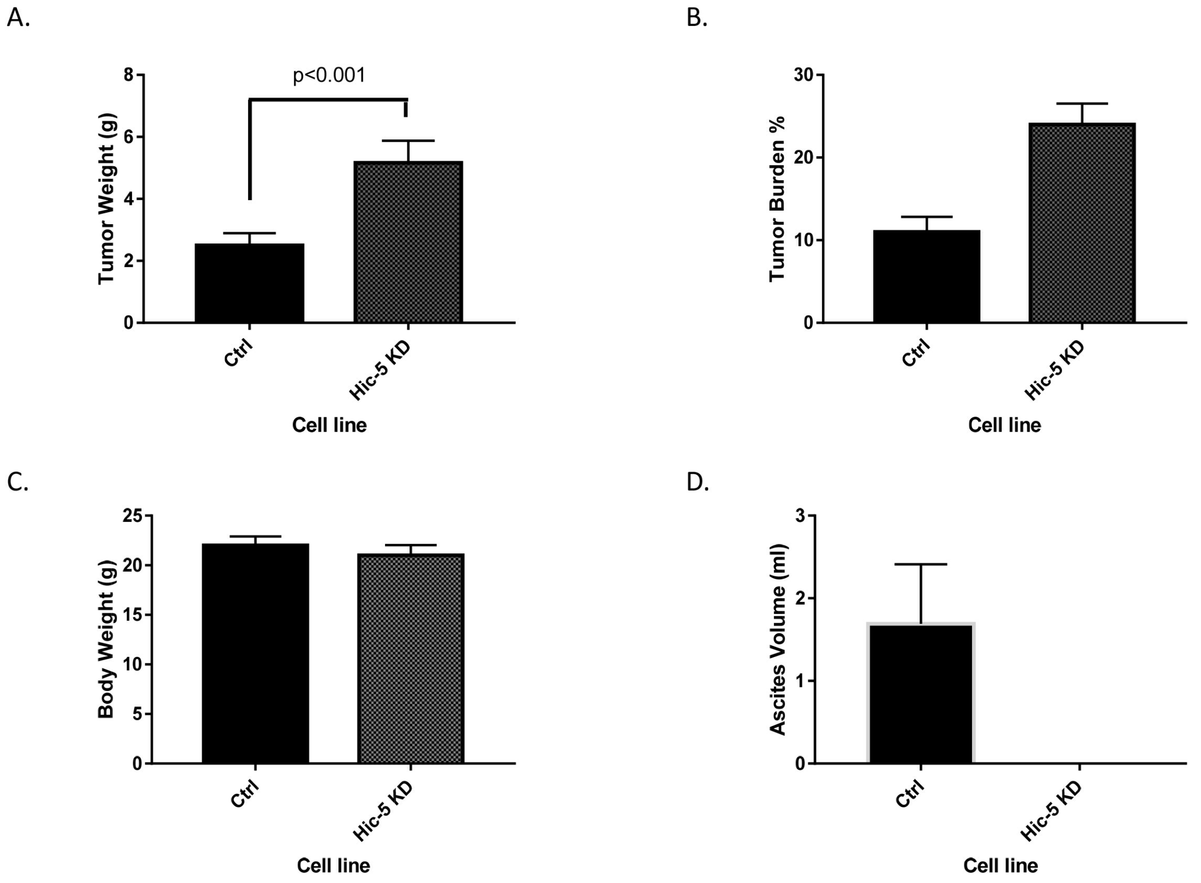

Supplementary Figure 8: (A) Tumor weight in SCID mice were measured. Data shown are the tumor weight averages with SD from 8 mice in mock.transfected control (Ctrl) and shRNA-Hic-5 knockdown (KD) injected mice. **(B)** Tumor burden in SCID mice were measured. Data shown are the % averages with SD from 8 mice in mock-transfected control (ctrl) and shRNA-Hic-5 knockdown (KD) injected mice. **(C)** Body weight in SCID mice were measured. Data shown are the body weight averages with SD from 8 mice in mock-transfected control (Ctrl) and shRNA-Hic-5 knockdown (KD) injected mice. **(D)** Ascites volumes were measured. Data shown are the ascites volume averages with SD from 8 mice in mock-transfected contol (Ctrl) and shRNA-Hic-5 knockdown (KD) injected mice.

Supplementary Table 1: Genes, differentially expressed in SKOV3 cells (≥ 2.0 fold, p≤0.05) following Hic-5 knockdown

See Supplementary File 1

Supplementary Table 2: Genes, differentially expressed in A2780s cells (≥ 1.5 fold, p≤0.05) following Hic-5 overexpression

See Supplementary File 2

Supplementary Table 3: Dilution and technique used for each antibody in IHC and Western blot analyses

Antibody	Species	Dilution TMA	Company	Retrieval	Incubation TMA	Dilution WB	Incubation WB
Hic-5	Rabbit	1:75	Aviva Systems Biology	Microwave	4 °C overnight	1:1000	4 °C overnight
E-cadherin	Rabbit	1:100	Santa Cruz	Microwave	4 °C overnight	1:1000	4 °C overnight
N-cadherin	Rabbit	1:100	Abcam	Microwave	4 °C overnight	1:1000	4 °C overnight
EPCAM	Mouse	N/A	Santa Cruz	N/A	4 °C overnight	1:1000	4 °C overnight
TWIST	Goat	N/A	Santa Cruz	N/A	4 °C overnight	1:1000	4 °C overnight
SNAIL	Rabbit	N/A	Santa Cruz	N/A	4 °C overnight	1:1000	4 °C overnight
vimentin	Mouse	N/A	Santa Cruz	N/A	4 °C overnight	1:2000	4 °C overnight
Paxillin	Mouse	1:75	BD Biosciences	Microwave	4 °C overnight	1:1000	4 °C overnight
p-FAK (Y397)	Rabbit	N/A	Cell Signaling	N/A	N/A	1:1000	4 °C overnight
FAK	Rabbit	N/A	Cell Signaling	N/A	N/A	1:1000	4 °C overnight
RhoA	Mouse	N/A	Abcam	N/A	N/A	1:500	4 °C overnight
Ki-67	Rabbit	1:100	Santa Cruz	Microwave	4 °C overnight	1:1500	4 °C overnight
DDK	Mouse	N/A	Origene	N/A	N/A	1:2000	4 °C overnight
Anti- β -Actin	N/A	N/A	Santa Cruz	N/A	N/A	1:2000	1 h/room temperature

Supplementary Table 4: Primers used for & MSP analysis and Quantitative PCR (qPCR) and RT-PCR.

See Supplementary File 3
