# Supplementary material for: Hic-5 regulates epithelial to mesenchymal transition in ovarian cancer cells in a TGFβ1-independent manner
Source: Oncotarget. 2017 Jul 31;8(47):82506–30. doi: 10.18632/oncotarget.19714 (PMC5669907; doi:10.18632/oncotarget.19714)
Supplement: Supplementary file 4 [file oncotarget-08-82506-s004.doc]

**Supplemental Table 4:** Primers used for & MSP analysis and Quantitative PCR (qPCR) and RT-PCR.

|  |  | **MSP Primers** |  | | |
| --- | --- | --- | --- | --- | --- |
| **Position** | **Primer** | **Primer Sequence** | **Product Size (bp)** | | |
| CDH1 Primer 1 (CpG 104) Methylation Outer | Forward | 5’- AGGGTTATCGCGTTTATGC -3’ | 297 | |  |
|  | Reverse | 5’- CCCATAACTAACCGAAAACG -3’ |  | |  |
| CDH1 Primer 1 (CpG 104) Nested no-Methylation Outer | Forward | 5’- TAGAGGGTTATTGTGTTTATGT -3’ | 297 | |  |
|  | Reverse | 5’- ACCCATAACTAACCAAAAACACC -3’ |  | |  |
| CDH1 Primer 1 (CpG 104) Methylation Inner | Reverse | 5’- CCGACGCCACTAAAAAAA -3’ | 179 | |  |
| CDH1 Primer 1 (CpG 104) Nested no-Methylation Inner | Reverse | 5’- TCCAACACCACTAAAAAAAAA -3’ | 179 | |  |
| CDH1 Primer 2 (CpG 104) Methylation Outer | Forward | 5’- GATTTGCGAGGGACGTATTC -3’ | 201 | |  |
|  | Reverse | 5’- AAAACAAAAAAACCGCGCT -3’ |  | |  |
| CDH1 Primer 2 (CpG 104) Nested no-Methylation Outer | Forward | 5’- TTTGATTTGTGAGGGATGTATTT-3’ | 201 | |  |
|  | Reverse | 5’- AAAAAAACAAAAAAACCACACT-3’ |  | |  |
| CDH1 Primer 2 (CpG 104) Methylation Inner | Reverse | 5’- ACTAAAAAATCCGAAACGCG -3’ | 151 | |  |
| CDH1 Primer 2 (CpG 104) Nested no-Methylation Inner | Reverse | 5’- ACTAAAAAATCCAAAACACACAA 3’ | 151 | |  |
|  |  | **qPCR Primers** |  |  | |
| **Gene** | **Primer** | **Primer Sequence** | **Product Size (bp)** | **Genbank Accession #** | |
| *Hic-5* | Forward | 5’- GGCCCCATCCTGGATAACTA -3’ | 252 | NM_001042454 | |
|  | Reverse | 5’- GCAGAAGGTGCATGTGAAGT -3’ |  |  | |
| *MET* | Forward | 5’- AAGAGGGCATTTTGGTTGTG -3’ | 136 | NM_000245.3 | |
|  | Reverse | 5’- GATGATTCCCTCGGTCAGAA -3’ |  |  | |
| *JAG1* | Forward | 5’- GACTCATCAGCCGTGTCTCA -3’ | 190 | NM_000214.2 | |
|  | Reverse | 5’- TGGGGAACACTCACACTCAA -3’ |  |  | |
| *TGFBR2* | Forward | 5’- CCAGAACCAAGCAGAGAAGG -3’ | 131 | NM_001024847 | |
|  | Reverse | 5’- GCTCCGAGTGGATGTAGAGC -3’ |  |  | |
| *FN1* | Forward | 5’- GAGAGAAGTGGGACCGTCAG -3’ | 165 | NM_001306129 | |
|  | Reverse | 5’- TGGCACCGAGATATTCCTTC -3’ |  |  | |
| *JUN* | Forward | 5’- CCCCAAGATCCTGAAACAGA -3’ | 168 | NM_002228.3 | |
|  | Reverse | 5’- CCGTTGCTGGACTGGATTAT -3’ |  |  | |
| *CDH2* | Forward | 5’- AGGATCAACCCCATACACCA -3’ | 125 | NM_001308176 | |
|  | Reverse | 5’- TGGTTTGACCACGGTGACTA -3’ |  |  | |
| *CDH18* | Forward | 5’- AGGATGTACGGGAGAACGTG -3’ | 119 | NM_001167667 | |
|  | Reverse | 5’- CCGGTACTTGAGCTCCTCAG -3’ |  |  | |
| *CXCL8* | Forward | 5’- AAGAAACCACCGGAAGGAAC -3’ | 123 | NM_000584.3 | |
|  | Reverse | 5’- ACTCCTTGGCAAAACTGCAC -3’ |  |  | |
| *GNG11* | Forward | 5’- TGCAGACACTTGGACCTCTG -3’ | 110 | NM_004126.3 | |
|  | Reverse | 5’- GTGGGCAAAGATTCACTGGT -3’ |  |  | |
| *CXCL1* | Forward | 5’- GAAAGCTTGCCTCAATCCTG -3’ | 107 | NM_001511.3 | |
|  | Reverse | 5’- CGTCCATAAAGAGCCTCGAC -3’ |  |  | |
| *BAMBI* | Forward | 5’- AAGCAGACCTCAGCAACGAT -3’ | 129 | NM_012342.2 | |
|  | Reverse | 5’- AAAGGCCTTCAAGCTGTTCA -3’ |  |  | |
| *EDN1* | Forward | 5’- CTTTGAGGGACCTGAAGCTG -3’ | 146 | NM_001955.4 | |
|  | Reverse | 5’- CTGTTTCTGGAGCTCCTTGG -3’ |  |  | |
| *TCF4* | Forward | 5’- CCGTCCAGGAACTATGGAGA -3’ | 171 | NM_001083962 | |
|  | Reverse | 5’- CTGGTGGCAACCCTGTAAGT -3’ |  |  | |
| *HMGA2* | Forward | 5’- GGCCAGCTCATAAAATGGAA -3’ | 190 | NM_001300918 | |
|  | Reverse | 5’- TACTGTTCCATTGGCCACAA -3’ |  |  | |
| *HGF* | Forward | 5’- CTGGTTCCCCTTCAATAGCA -3’ | 168 | NM_000601.5 | |
|  | Reverse | 5’- CTCCAGGGCTGACATTTGAT -3’ |  |  | |
| *CDO1* | Forward | 5’- ACCCCACAGATCTGAACCTG -3’ | 127 | NM_001323565 | |
|  | Reverse | 5’- GTGCCGAAACGTAAGGATGT -3’ |  |  | |
| *NR2F1* | Forward | 5’- TACGTGAGGAGCCAGTACCC -3’ | 122 | NM_005654.5 | |
|  | Reverse | 5’- CCTACCAAACGGACGAAGAA -3’ |  |  | |
| *SPRY2* | Forward | 5’- CTAAGCCTGCTGGAGTGACC -3’ | 191 | NM_001318536 | |
|  | Reverse | 5’- GTGTTTCGGATGGCTCTGAT -3’ |  |  | |
| *DDIT3* | Forward | 5’- GCGCATGAAGGAGAAAGAAC -3’ | 165 | NM_001195053 | |
|  | Reverse | 5’- CCAATTGTTCATGCTTGGTG -3’ |  |  | |
| *SNAI1* | Forward | 5’- CCAATCGGAAGCCTAACTACAG -3’ | 155 | NM_005985.3 | |
|  | Reverse | 5’- GACAGAGTCCCAGATGAGCATT -3’ |  |  | |
| *TRIB3* | Forward | 5’- CCACTACCCCTTCCAGGACT -3’ | 249 | NM_001301188 | |
|  | Reverse | 5’- AGTCCATCAGGGACCACCT -3’ |  |  | |
| *SPP1* | Forward | 5’- TGAAACGAGTCAGCTGGATG -3’ | 162 | NM_000582.2 | |
|  | Reverse | 5’- TGAAATTCATGGCTGTGGAA -3’ |  |  | |
| *PCDH17* | Forward | 5’- CAGGCTGACAGTGACCAAGA -3’ | 164 | NM_001040429 | |
|  | Reverse | 5’- TCAGAATGACCAAGCACTCG -3’ |  |  | |
| *ANXA1* | Forward | 5’- GCAGGCCTGGTTTATTGAAA -3’ | 203 | NM_000700.2 | |
|  | Reverse | 5’- GCTGTGCATTGTTTCGCTTA -3’ |  |  | |
| *MMP10* | Forward | 5’- AATGAGTTCTGGGCCATCAG -3’ | 151 | NM_002425.2 | |
|  | Reverse | 5’- ATTTGTCCGCTGCAAAGAAG -3’ |  |  | |
| *GUSB* | Forward | 5’- ATACGTGGTTGGAGAGCTCATT -3’ | 187 |  | |
|  | Reverse | 5’- CTTGGCTACTGAGTGGGGATAC -3’ |  |  | |
| *18S* | Forward | 5’-AACCCGTTGAACCCCATT-3’ | 119 |  | |
|  | Reverse | 5’-CCATCCAATCGGTAGTAGCG-3’ |  |  | |

Note: Forward and reverse primers were designed to recognize different exons of the target gene. The primer positions are based on the reference sequence of the target genes in the Genbank. Expression of 18S and GUSB was used as control for sample normalization in quantification of the gene of interest expression by real-time RT-PCR analysis and qPCR.
